# Organization of Astrocytic GLT‐1 at Cortical Inhibitory Synapses

**DOI:** 10.1002/glia.70180

**Published:** 2026-06-07

**Authors:** Marcello Melone, Michael Di Palma, Annalisa Scimemi, Fiorenzo Conti

**Affiliations:** ^1^ Section of Neuroscience and Cell Biology, Department of Experimental and Clinical Medicine Università Politecnica Delle Marche Ancona Italy; ^2^ Center for Neurobiology of Aging IRCCS INRCA Ancona Italy; ^3^ Department of Biology SUNY Albany Albany New York USA

**Keywords:** astrocytes, astrocytic leaflets, cerebral cortex, glutamate spillover, glutamate transporters, inhibitory synapses, synaptic microenvironment

## Abstract

Glutamate spillover from excitatory synapses modulates neighboring inhibitory synapses, yet the ultrastructural organization of the major glutamate transporter GLT‐1 at these sites remains poorly defined. Using quantitative pre‐embedding electron microscopy in rat and human cortex, we found that GLT‐1‐positive astrocytic leaflets (ALs) were frequently juxtaposed to morphologically identified symmetric synapses, with similar prevalence across axo‐somatic, proximal axo‐dendritic, and distal axo‐dendritic subtypes. Because inhibitory synapses are embedded in a dense excitatory neuropil, we applied distance‐based phenotyping relative to the nearest asymmetric synapse to define symmetric‐associated GLT‐1+ ALs. Within this population, distal axo‐dendritic symmetric synapses showed shorter AL‐to‐synaptic‐edge distances and were embedded in a tighter local excitatory microenvironment. Post‐embedding immunogold further showed that GLT‐1 was enriched at the plasma membranes of ALs and localized extrasynaptically relative to symmetric synapses. Consistently, symmetric‐associated membrane GLT‐1 and closely spaced GLT‐1/α2 couples (with an interdistance ≤ 50 nm) were preferentially localized within 1000 nm of distal symmetric synapses compared to proximal. Similar organizational features of membrane GLT‐1/α2 couples were observed in human cortex. These findings identify a subtype‐dependent extrasynaptic astrocytic GLT‐1 organization at cortical inhibitory synapses and provide a morphological framework for glutamate‐dependent modulation of inhibitory signaling.

## Graphical abstract

- GLT‐1+ ALs frequently juxtapose symmetric synapses.
- Distance‐based phenotyping identifies symmetric‐ and asymmetric‐associated GLT‐1+ ALs.
- Distal inhibitory synapses show enriched GLT‐1/α2 couples in ALs within 1000 nm, conserved in human cortex.

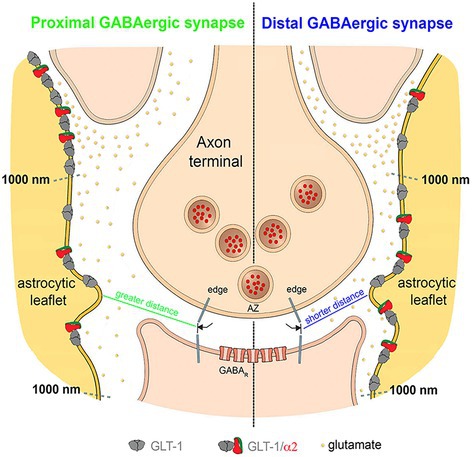

## 1 Introduction

Glutamate, the main excitatory transmitter in cerebral cortex (Conti and Weinberg 1999), mediates point‐to‐point synaptic transmission, but also spills out of the synaptic cleft of active excitatory synapses to neighboring synapses, thereby exerting modulating effects at farther sites (Isaacson 2000; Kullmann 2000; Farrant and Nusser 2005; Semyanov 2005; Herman et al. 2011; Reiner and Levitz 2018). Such modulation, not predictable on anatomical grounds, occurs at neighboring excitatory and inhibitory synapses through glutamate homo‐ and hetero‐receptors, respectively (Lujan et al. 2005; Pinheiro and Mulle 2008; Reiner and Levitz 2018).

Glutamate spillout from excitatory synapses and extracellular glutamate levels are closely regulated through high‐affinity plasma membrane transporters, of which GLT‐1 is the most abundantly expressed in most brain regions (Conti and Weinberg 1999; Danbolt 2001). At glutamatergic synapses, astrocytic GLT‐1 modulates glutamate spillout from synaptic cleft to extrasynaptic space (Clements 1996; Diamond and Jahr 1997; Otis and Jahr 1998; Huang and Bergles 2004; Herman and Jahr 2007; Murphy‐Royal et al. 2015). Experimental data on the expression of GLT‐1 at central inhibitory synapses are scanty: GLT‐1 blockers modulate GABAergic transmission through increased glutamate spillout targeting glutamate hetero‐receptors (Semyanov and Kullmann 2000; Mitchell and Silver 2000; Piet et al. 2003; Scimemi et al. 2004; Drew et al. 2008; Bonfardin et al. 2010; Petroccione et al. 2025), and early localization studies using confocal microscopy showed that GLT‐1 is in close proximity to inhibitory GABAergic synapses (Minelli et al. 2001). In addition, it is worth noting that recent ultrastructural studies indicate that thin astrocytic leaflets (ALs), the fine peripheral astrocytic processes that contact synaptic elements (Semyanov and Verkhratsky 2021; Pietrobon and Conti 2024; Verkhratsky et al. 2025), are frequently found in proximity to both excitatory and inhibitory synapses and can affect multiple neighboring synapses (Kikuchi et al. 2020; Benoit et al. 2025).

In this context, a critical pending issue is how GLT‐1‐positive ALs positioned near morphologically identified symmetric synapses relate to the immediately surrounding excitatory synapse neighborhood, given the dense packing of asymmetric synapses in cortical neuropil. Disambiguating this shared microenvironment is essential to interpret astroglial GLT‐1 positioning at inhibitory synapses in morphological terms relevant to excitatory–inhibitory crosstalk.

On this basis, we investigated the organization of GLT‐1 at cortical GABAergic synapses in rats and humans using quantitative pre‐ and post‐embedding electron microscopy. We quantified the prevalence of GLT‐1‐positive ALs juxtaposed to morphologically identified symmetric synapses, and we explicitly measured astroglial geometry relative to both the symmetric synapse and the nearest neighboring asymmetric synapse within the same ultrastructural field, providing a conservative framework to interpret GLT‐1 positioning within the local asymmetric (i.e., excitatory) synaptic microenvironment. We further assessed membrane‐associated GLT‐1 and its close spatial relationship with the astrocytic Na^+^/K^+^‐ATPase α2 (hereafter referred to as α2) subunit using post‐embedding immunogold electron microscopy and examined whether key organizational features are conserved in human cortex.

## 2 Materials and Methods

### 2.1 Tissue Preparation

Male adult albino rats (150–350 g; *n* = 15; Sprague–Dawley) were used. Their care and handling were approved by the local Institutional Committee for Animal Research of Università Politecnica delle Marche (approval AN.40A31.N.FV0). Experiments were performed in line with European Community Council Directives 86/609, 2010/63/EU. Animals were anesthetized with an intraperitoneal injection of chloral hydrate (300 mg/kg) and perfused transcardially with a flush of saline solution, followed by 4% freshly depolymerized paraformaldehyde (PB; pH 7.4). Brains were removed, post‐fixed in the same fixative for 6 days, and cut on a vibratome in 50 μm serial parasagittal sections, which were collected in PB until processing. For human studies, we used samples of cortical tissue from two surgical specimens employed in a previous study (Melone et al. 2011; cases # HBC 980510, male, 60 years old, cytoarchitectonic area 46; # HBC 030129, male, 64 years old, cytoarchitectonic area 8; both cases were affected by frontal meningioma with high intracranial pressure as major symptom; both were under treatment with valproate 1000 mg/day for 30 days). The cortical tissue was not taken from the area surrounding the tumors; it consisted of macroscopically normal tissue that had to be resected to access deep‐seated tumors, or that was part of “tactical lobectomies”. Tissue showed no signs of edema, and none of the patients experienced preoperative or postoperative seizures. Informed consent for the surgical procedure was obtained from all patients. Specimens were quickly immersed for 2–3 min in a cold solution of 4% PFA in PB for 2–3 h. They were then transferred to fresh 4% PFA solution and stored for 24–48 h at 4°C. The samples were cut into small blocks, which underwent post‐fixation for an additional 24–72 h at 4°C in the same solution. After being washed with PB, the samples were stored at −20°C in a solution containing 30% glycerol, 30% ethylene glycol, 30% distilled water, and 10% PB (0.4 M). Before sectioning, the blocks were washed several times in PB and then cut into 50 μm thick sections using a vibratome (Pelco easislicer, Ted Pella, CA, USA).

### 2.2 Pre‐Embedding Electron Microscopy

After treatment with H_2_O_2_ (1% in PB; 30 min), to remove endogenous peroxidase activity, sections were rinsed in PB, incubated in 10% NDS (1 h) in PB and then in a solution containing GLT‐1 or α2 primary antibodies (Table 1 for details and dilutions) for 2 h at room temperature and subsequently overnight at 4°C. Sections were rinsed in PB and incubated for 20 min in 10% NDS in PB and then for 90 min in a solution containing biotinylated secondary antibodies (Table 1). They were subsequently rinsed in PB, incubated in avidin‐biotin peroxidase complex (ABC Elite PK6100, Vector), washed in PB, and incubated in 3,3′‐diaminobenzidine tetrahydrochloride (DAB; 0.08% in 0.05 M Tris buffer, pH 7.6 with 0.06% H_2_O_2_). Sections were postfixed in 1% osmium tetroxide in PB for 1 h and contrasted with 1% uranyl acetate in maleate buffer (pH 6.0; 1 h) as described previously (Melone et al. 2009, 2019). Dehydrated sections were immersed in propylene oxide, infiltrated with a mixture of Epon/Spurr resins (Electron Microscopy Sciences), sandwiched between Aclar films, and polymerized at 60°C for 48 h. For rat studies, small blocks of tissue containing layers II/III of the first somatosensory cortex (SI) were selected by light microscopic inspection based on the typical features of rat parietal cortex (as conspicuous layer IV with intermingled dysgranular regions, densely packed layers II/III, and a relatively cell‐free layer Va [Chapin and Lin 1990]). We chose to focus on layers II/III of SI because we have previously investigated the cellular, subcellular, and synaptic localization of GLT‐1 and α2 in this cortical region (Minelli et al. 2001; de Vivo et al. 2010; Melone et al. 2009, 2019) and because layers II/III contain a large number of glutamatergic and GABAergic synapses (DeFelipe et al. 1999). Chips were glued to blank epoxy and sectioned with an ultramicrotome (MTX and PTXL; RMC, Boeckeler, Tucson, AZ, USA). The most superficial ultrathin sections (~60 nm) were mounted on 200‐mesh copper grids, stained with Sato's lead and examined with a CM10 and Philips EM 208 electron microscope coupled to a MegaView‐II high‐resolution CCD camera (Soft Imaging System). Identification of labeled and unlabeled profiles was based on established morphological criteria (Peters et al. 1991).

**TABLE 1:** Primary and secondary antibodies.

Protein	Source	Primary antibody	Host	Characterized in	Dilution	Secondary antibody	Source	Dilution
GLT‐1a	JD Rothstein	GLT‐1a^a^ RRID:AB_2314565	R	Rothstein et al. (1994) Chen et al. (2004) Omrani et al. (2009) Melone et al. (2011)	1.0 μg/mL (IP) 2.3 μg/mL (IG)	Biotinylated 12 nm gold	Vector Jackson	1:150 1:20
GLT‐1a	Merck	AB1783^b^ RRID:AB_90949	GP	Figiel and Engele (2000) Chung et al. (2008) Melone et al. (2019)	1:250 (IG)	18 nm gold	Jackson	1:20
α2‐ATPase	Merck	AB07‐674^c^ RRID:AB_390164	R	Melone et al. (2019)	1:150 (IP) 1:60 (IG)	Biotinylated 12 nm gold	Vector Jackson	1:150 1:20

Abbreviations: GP, guinea pig; IG, immunogold for post‐embedding electron microscopy; IP, immunoperoxidase for pre‐embedding electron microscopy; R, rabbit.

^a^ Raised against a synthetic peptide corresponding to AA 559–573 (SADCSVEEEPWKREK) of GLT‐1a rat C‐terminus (Rothstein et al. 1994). In Western blots of rat brain homogenates, it recognized a GLT‐1‐immunoreactive band with a molecular mass of ~70 kDa; immunoreactivity was completely abolished when antibodies were preadsorbed with synthetic GLT‐1 peptide (Rothstein et al. 1994). In GLT‐1 knock‐out mice, GLT‐1a immunoreactivity was totally absent in Western blots of brain lysates and in sections processed for IP light microscopy and pre‐embedding electron microscopy (Chen et al. 2004). In GLT‐1 knock‐out mice (Tanaka et al. 1997), post‐embedding IG detection of GLT‐1a was absent (Omrani et al. 2009). In human cortical tissue GLT‐1 immunoreactivity was completely abolished by preincubation of GLT‐1a antibodies with 10^−3^ M of AA 559–573 synthetic peptide; Western blots of cortical synaptic membranes confirmed that GLT‐1a antibodies recognized a band of ∼70 kDa (Melone et al. 2011).

^b^ Raised against a synthetic peptide corresponding to AA 554–573 (AANGKSADCSVEEEPWKREK) of GLT‐1a rat C‐terminus. In Western blots of proteins from primary glial cell cultures of rat cortex (Figiel and Engele 2000) and of rat striatal and substantia nigra homogenates (Chung et al. 2008), it recognized a single band of ~70 kDa corresponding to the molecular weight of GLT‐1; preadsorption with the synthetic GLT‐1a peptide completely abolished immunostaining (manufacturer's technical information). Double‐labeling fluorescence with the antibody characterized by JD Rothstein demonstrated a virtually complete colocalization of fluorescent signals in cortical sections (data not shown). Immunogold electron microscopic analysis showed that in GLT‐1 knock‐out mice (Tanaka et al. 1997) immunoreactivity was abolished (Melone et al. 2019).

^c^ Raised against a synthetic peptide corresponding to AA 432–445 of human α2 NKA (CKAGQENISVSKRDT; sequence is identical in rat and mouse). On WB of rat brain microsomal preparation, it recognizes a band of ~105 kDa (manufacturer's technical information); on WB of rat cortical synaptic membranes, it recognizes a single band of ~100 kDa (Melone et al. 2019). On IP of fixed rat kidney (where α2 isoform is particularly low) immunoreactivity was virtually absent, and immunoreactivity in both IP and WB α2 was totally undetectable by pre‐adsorbing α2 antibodies with 10^−3^ M synthetic peptide corresponding to AA 432–445 (lot number CR‐04‐00390, Sigma; Melone et al. 2019). Furthermore, WB and immunofluorescence of cerebral cortex from adult FHM2 heterozygous knock‐in mice carrying the human W887R mutation in the *Atp1a2* orthologous gene, reveal a significant reduction of α2 protein detection compared to wild type mice (Melone et al. 2019).

### 2.3 Data Collection and Analysis

Microscopic fields used for GLT‐1 and α2 localization at symmetric synapses were randomly selected and captured at an original magnification of 30,000 or 50,000× (Melone et al. 2019). Symmetric synapses were identified ultrastructurally according to established criteria (Peters et al. 1991; DeFelipe et al. 1999). Specifically, a symmetric synapse was recognized by a vesicle‐containing presynaptic terminal, a clearly identifiable synaptic cleft, and a well‐defined active zone/postsynaptic density (AZ/PSD) complex with a thin/symmetric PSD, allowing discrimination from asymmetric synapses characterized by a prominent PSD (Tyler and Pozzo‐Miller 2001). Astrocytic processes in the immediate perisynaptic neuropil were identified by their typical irregular outlines and characteristic cytological features, including ribosomes, glycogen granules, occasional fibril bundles, and sparse cytoplasmic organelles (Peters et al. 1991). In line with the terminology commonly used to describe thin perisynaptic astrocytic profiles, we refer to these processes as ALs (Semyanov and Verkhratsky 2021; Pietrobon and Conti 2024; Verkhratsky et al. 2025). According to different post‐synaptic targets of interneurons, immuno‐positive ALs of symmetric synapses were sampled at axo‐somatic, proximal, distal, or spinous axo‐dendritic synapses (dendrites were considered distal if their diameter was ≤ 1 μm and proximal if it was > 1 μm) (DeFelipe 2002; Mariotti et al. 2018; Ascoli et al. 2008; Melone et al. 2014; Somogyi et al. 1998). To quantify the prevalence and spatial relationship of GLT‐1– or α2–positive (+) ALs relative to symmetric synapses, we adopted an explicit operational definition of synapse–astroglia association. An AL+ was considered associated with a symmetric synapse when it was juxtaposed to one or both neuronal domains of the symmetric synapse in the plane of section. Here, “juxtaposition” indicates an AL positioned close to one or both synaptic domains without implying direct membrane‐to‐membrane contact, membrane continuity, or a requirement for a minimal surface area of contact (see, for example, distance‐based formulations of synapse–astroglia spatial juxtaposition in Medvedev et al. 2014). Using this criterion, we quantified the proportion of ultrastructurally identifiable symmetric synapses exhibiting juxtaposed GLT‐1+ or α2+ ALs and performed the distance‐based AL phenotyping and local‐excitatory microenvironment analyses.

#### 2.3.1 Prevalence Analysis of Symmetric Synapses Juxtaposed to ALs+

Electron micrographs from pre‐embedding material were analyzed using FIJI (ImageJ, Schneider et al. 2012) after spatial calibration. Within each microscopical field, all morphologically identifiable symmetric synapses were first recognized based on established ultrastructural criteria reported above. Each symmetric synapse was then scored as + when an immunoreactive AL was juxtaposed to one or both neuronal synaptic domains (presynaptic and/or postsynaptic specialization) in the plane of section, according to the operational definition reported above. Symmetric synapses lacking juxtaposed ALs + were scored as negative. All symmetric synapses within the field were manually annotated and recorded in a spreadsheet, including both positive and negative cases. Symmetric synapses were further subclassified according to postsynaptic target into axo‐somatic (Axo‐Som), proximal axo‐dendritic (Axo‐Den), and distal axo‐dendritic (Axo‐den) subtypes. Within the subset of symmetric synapses juxtaposed to ALs+, astrocytic leaflets were additionally annotated as shared or not shared, where shared denoted an AL+ juxtaposed to the symmetric synapse and also juxtaposed to a neighboring asymmetric synapse within the same field. This prevalence and sharing annotations provided the basis for the subsequent distance‐based phenotyping described in the next subsection.

#### 2.3.2 Distance‐Based Phenotyping of ALs + Relative to Symmetric Versus Nearby Asymmetric Synapses

For ALs + symmetric synapses (as defined above), we quantified the spatial relationship between the juxtaposed AL and (i) the symmetric synapse it was juxtaposed with, and (ii) the nearest neighboring asymmetric synapse within the same electron microscopical field. As above, measurements were performed in FIJI (ImageJ) using the polyline tool to trace membrane‐following distances along the AL profile. Specifically, we measured the distances between each symmetric (AL‐Dsym) and the nearest asymmetric (AL‐Dasym) edge of AZ/PSD complexes and the closest point of ALs + process passing along the membrane of profiles (Parker et al. 2021). These paired measurements were used to compute a proximity phenotype: Δ = AL‐Dasym – AL‐Dsym, where Δ > 0 indicates that the AL+ was closer to the symmetric synapse than to the nearest asymmetric synapse (‘AL symmetric‐associated’), whereas Δ < 0 indicates that the AL is closer to the nearby asymmetric synapse (“AL asymmetric‐associated”). This Δ‐based phenotyping was subsequently used to select the symmetric‐associated ALs population for downstream comparisons.

#### 2.3.3 Local Excitatory Microenvironment Analysis

To quantify the local excitatory microenvironment surrounding symmetric synapses bearing symmetric‐associated GLT‐1+ ALs, as defined in the previous subsection, we implemented a custom FIJI/ImageJ macro (available upon request) that adapts a Sholl‐like radial analysis to single‐section pre‐embedding images. In particular, this workflow adapts the general principle of radial spatial binning, originally developed and applied in three‐dimensional ultrastructural reconstructions of synaptic organization (Schikorski and Stevens 1997) and synapse–astroglia relationships (Ventura and Harris 1999), to a conservative two‐dimensional nearest‐neighbor spatial reference framework. The purpose of this two‐dimensional framework was to contextualize GLT‐1+ ALs geometry relative to the local excitatory neighborhood, consistent with microenvironment‐based perisynaptic glial organization (Rusakov 2001). For each analyzed field, the synaptic contact of the symmetric synapse was traced using the polyline tool. The macro automatically computed the curvilinear midpoint of this traced synaptic contact, which was used as the reference center for Euclidean measurements and for generating radial masks. Within the same field, the nearest neighboring asymmetric synapse was identified ultrastructurally and manually annotated using the multi‐point tool (single point). The macro computed the Euclidean distance from the symmetric synaptic‐contact midpoint to this annotated asymmetric synapse and returned this value as the nearest‐neighbor distance (sym–asym distance). Concentric radial masks centered on the symmetric synaptic‐contact midpoint were generated in 250‐nm steps and used to provide a standardized two‐dimensional spatial reference of the relative position of the nearest asymmetric synapse with respect to the symmetric contact. Quantitative analyses and statistical comparisons focused on nearest‐neighbor distances within the submicron range (0–1.0 μm), consistent with the dense packing of excitatory synapses in cortical neuropil (Antón‐Sánchez et al. 2014).

### 2.4 Post‐Embedding Electron Microscopy

Sections were processed with an osmium‐free embedding method (Phend et al. 1995). Dehydrated sections were immersed in propylene oxide, infiltrated with a mixture of Epon/Spurr resins, sandwiched between Aclar films, and polymerized at 60°C for 48 h. After polymerization, chips including layers II/III were cut from the wafers, glued to blank resin blocks, and sectioned with an ultramicrotome. Ultrathin sections (60–80 nm) were mounted on 300‐mesh nickel grids and processed for immunogold labeling (Phend et al. 1995; Melone et al. 2019). Briefly, after treatment with 4% para‐phenylenediamine in Tris‐buffered saline (0.1 M Tris, pH 7.6, with 0.005% Tergitol NP‐10 [TBST]), grids were washed in TBST (pH 7.6), transferred to 1% NDS in TBST (pH 7.6) for 15 min, and then incubated overnight in TBST (pH 7.6) containing anti‐GLT‐1 primary antibodies for single labeling or a mixture of anti‐GLT‐1 and anti‐α2 primary antibodies for double immunogold studies (see Table 1 for dilutions). Grids were washed in TBST (pH 8.2), transferred for 10 min to 1% NDS in TBST (pH 8.2), incubated for 2 h in TBST (pH 8.2) containing secondary antibodies conjugated to gold particles (Table 1), washed in distilled water, and finally stained with uranyl acetate and Sato's lead. The optimal concentration of the primary antibodies was established by testing several dilutions; the chosen concentration was the one with the lowest background that still resulted in labeling (Melone et al. 2009, 2019). Gold particles were not detected when the primary antiserum was omitted. When normal serum was substituted for the immune serum, sparse and scattered gold particles were detected but none showed a specific relationship with the astrocytic processes.

### 2.5 Data Collection and Analysis

Ultrathin sections were examined at 50,000–85,000×; fields that included at least one immunolabeled profile, that is, a symmetric synapse juxtaposed to AL, were acquired. Synapses were selected if they had a clear presynaptic AZ/PSD complex (Peters et al. 1991; Tyler and Pozzo‐Miller 2001). To determine the relative density of GLT‐1 in subcellular compartments, we identified the astrocytic profiles and pyramidal cell nuclei, counted the gold particles within labeled structures, and computed areas with FIJI/ImageJ. The background was calculated by estimating labeling density over pyramidal cell nuclei (Rácz et al. 2004; Melone et al. 2019). Particle densities were counted and compared to background labeling. Gold particles were considered as being associated with the plasma membrane if they were within 20 nm of the extracellular side of the membrane, and cytoplasmic if they were > 25 nm of the extracellular side of the membrane. In all subsets of symmetric synapses, the spatial position of membrane‐associated gold particles relative to synaptic specializations was quantified following established post‐embedding approaches (Kharazia and Weinberg 1999; Valtschanoff and Weinberg 2001; Rácz et al. 2004; Melone et al. 2019). Briefly, for each membrane‐associated GLT‐1 gold particle localized on AL juxtaposed to a symmetric synapse, the lateral position was defined as the distance along the astrocytic plasma membrane from the AZ/PSD edge to the center of the particle and measured in FIJI/ImageJ. Because ALs can be located near both symmetric and neighboring asymmetric synapses within the same field, for each particle we additionally measured the distance along the astrocytic membrane from the particle to the nearest neighboring asymmetric synaptic edge in the same field and computed a proximity phenotype Δ = (particle‐Asym) − (particle‐Sym), where particle‐Sym indicates the distance to the symmetric synaptic edge and particle‐Asym the distance to the neighboring asymmetric synaptic edge. Particles with Δ > 0 were classified as symmetric‐associated (i.e., closer to the symmetric than to the neighboring asymmetric contact) and were used for downstream distributional analyses consistently with the phenotyping approach used in pre‐embedding analyses. Accordingly, distributions of GLT‐1 particle distances from the symmetric synaptic edge were summarized in 200‐nm bins along the membrane using the symmetric‐associated (Δ > 0) particle subset. In line with the proximity phenotype criterion used in pre‐ and post‐embedding GLT‐1+ ALs analyses, in double‐labeled GLT‐1/α2+ ALs at proximal and distal symmetric synapses, the distance from the GLT‐1‐coding particle of the couple was used as reference for defining the symmetric‐associated GLT‐1/α2 couples. Next, all the interdistances of the symmetric‐associated GLT‐1 and α2 gold particles detected at the ALs membranes were collected and summarized in 200‐nm bins (Mariotti et al. 2018; Melone et al. 2019). Finally, symmetric‐associated GLT‐1/α2 couples with an edge‐to‐edge interparticle distance ≤ 50 nm (hereafter referred to as closely spaced couples), a criterion used in previous studies as indicative of close molecular proximity (Storey et al. 2012; Amiry‐Moghaddam and Ottersen 2013; Mariotti et al. 2018; Melone et al. 2019), were sublocalized in relation to symmetric and asymmetric synaptic contacts.

### 2.6 Statistical Analysis

All analyses were performed in R (RStudio) and Python using a custom scripted pipeline. Tests were two‐sided with *α* = 0.05. Non‐parametric tests were used for distance‐based variables. For multiple comparisons, Holm correction was applied to post hoc procedures (e.g., Dunn–Holm following Kruskal–Wallis tests; Fisher–Holm for pairwise comparisons of proportions). The unit of analysis depended on the endpoint and is specified in the relevant Methods subsections and in figure legends. For pre‐embedding datasets, proportions were compared using exact contingency‐table tests (Fisher/Fisher–Freeman–Halton). Paired within‐profile distance comparisons used Wilcoxon matched‐pairs tests. Across‐group comparisons of distances used Kruskal–Wallis with Holm‐adjusted post hoc tests. Associations between distance variables were assessed using Spearman correlation and robust trend estimation (Theil–Sen). For analyses involving radial masks, boundary coverage was quantified as the fraction of the theoretical circular area at the outer reference radius that was visible within the image frame. Fields were required to have ≥ 60% visible area at this outer radius; no fields were excluded, and the median visible‐area fraction was 0.89, indicating high boundary coverage around the symmetric synapse reference point. For post‐embedding datasets, particle/couple distance distributions were analyzed using non‐parametric tests (e.g., Mann–Whitney and distribution‐shape tests such as Kolmogorov–Smirnov), and proportions were compared using Fisher's exact tests. Unimodality of particle‐distance distributions was assessed using Hartigan's dip test when indicated. Detailed statistical outputs (test choice, *n*, and exact *p* values) are reported in the corresponding figure legends.

## 3 Results

### 3.1 GLT‐1 Is Widely Expressed in ALs Juxtaposed to Symmetric Synapses

**FIGURE 1:**
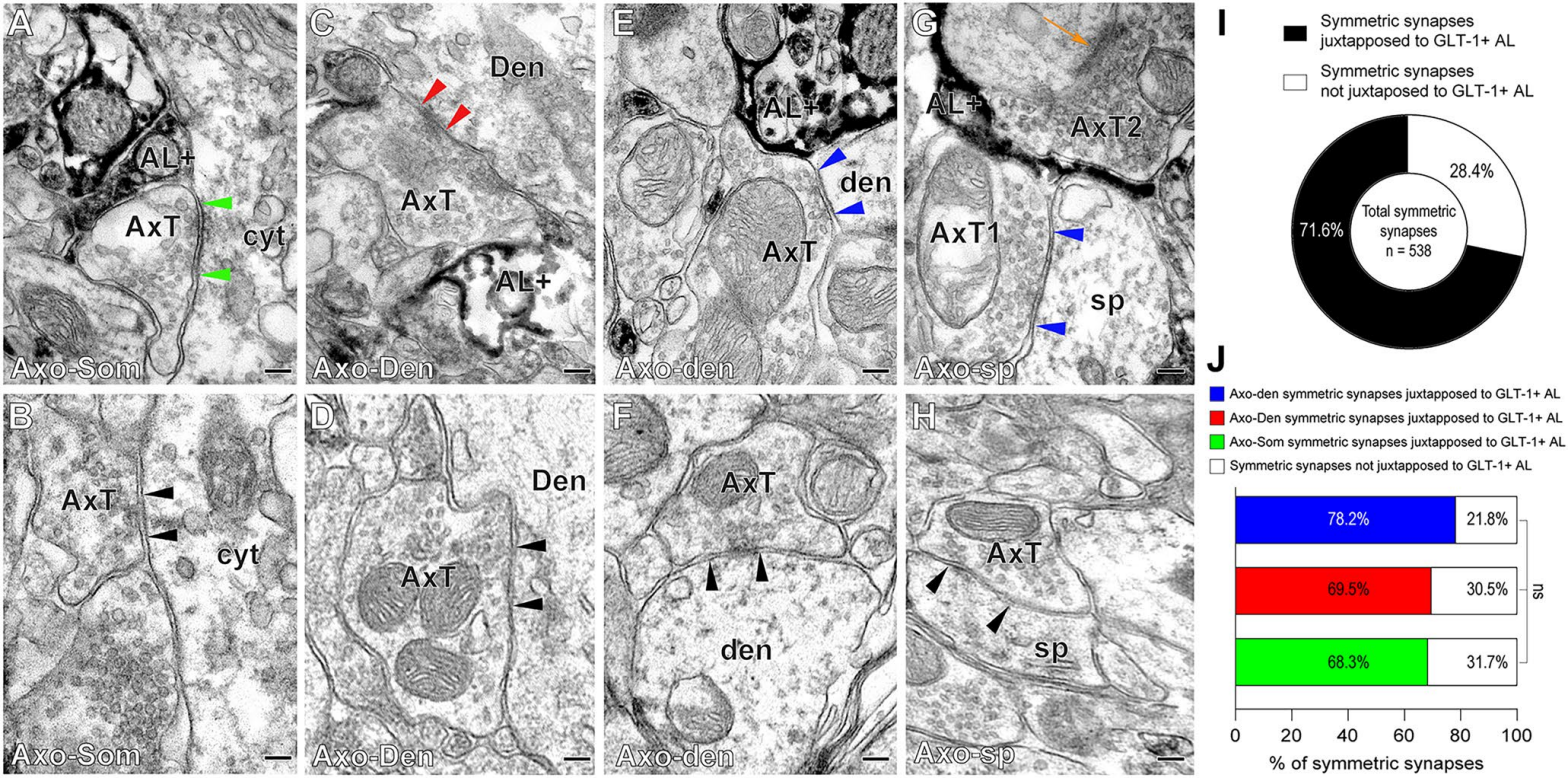
Prevalence of symmetric synapses juxtaposed to GLT‐1+ ALs. (A, C, E, G) Pre‐embedding electron microscopy of symmetric synapses (A, axo‐somatic, Axo‐Som; C, proximal axo‐dendritic, Axo‐Den; E, distal axo‐dendritic, Axo‐den, and G, axo‐spinous, Axo‐sp) showing neuronal synaptic domains juxtaposed to GLT‐1+ astrocytic leaflets (ALs+) containing dark immunopositive electron dense products. Colored arrowheads indicate symmetric synaptic edges; AxT, axon terminal forming a symmetric synaptic contact; cyt, neuronal cytoplasm; Den, proximal dendrite; den, distal dendrite; sp., spine. In G, AxT1 identifies the axon terminal of the symmetric synapse while AxT2 forms an asymmetric synaptic contact (marked by orange arrow). (B, D, F, H) Representative Axo‐Som, Axo‐Den, Axo‐den, and Axo‐sp symmetric synapses without juxtaposition to GLT‐1+ ALs. Black arrowheads indicate symmetric synaptic edges. (I) Positivity rate (386 of 538; 71.6%) of symmetric synapses with juxtaposed GLT‐1+ ALs. (J) The proportion of symmetric synapses juxtaposed to GLT‐1+ ALs was comparable among the different subtypes of symmetric synapses (68.3%, for Axo‐Som; 69.5% for Axo‐Den, and 78.2% for Axo‐den/sp.; Fisher–Freeman–Halton exact test, *p* = 0.069). Data were obtained from 12 ultrathin sections per animal, 3 animals. Scale bars: 80 nm.

We first investigated the subcellular localization of GLT‐1 in all morphologically identifiable GABAergic symmetric synapses using quantitative pre‐embedding electron microscopy. Our examination of randomly selected symmetric synapses of layers II/III in rat SI revealed that GLT‐1 is localized to ALs juxtaposed to synaptic domains in 386 out of 538 identifiable synapses, corresponding to 71.6% (Figure 1A–I). GABAergic synapses can be differentiated based on their post‐synaptic domains (Somogyi et al. 1998; DeFelipe et al. 2002; Ascoli et al. 2008; Mariotti et al. 2018). Thus, we investigated whether the presence of GLT‐1+ ALs varied among axo‐somatic (Axo‐Som, Figure 1A,B,J), proximal axo‐dendritic (Axo‐Den, Figure 1C,D,J), and distal axo‐dendritic (including both postsynaptic distal dendrites and spines, Axo‐den, Figure 1E–HJ) GABAergic synapses. The positivity rates for these three synaptic subtypes were 68.3% for Axo‐Som (*n* = 155 of 227), 69.5% for Axo‐Den (*n* = 98 of 141), and 78.2% for Axo‐den (*n* = 133 of 170), and their comparison indicated that GLT‐1+ ALs were similar among the different synapse subtypes (Fisher's exact test, *p* = 0.069; Figure 1J).

### 3.2 Distance‐Based Phenotyping Distinguishes Symmetric‐Associated GLT‐1+ ALs From Neighboring Asymmetric Synapses

**FIGURE 2:**
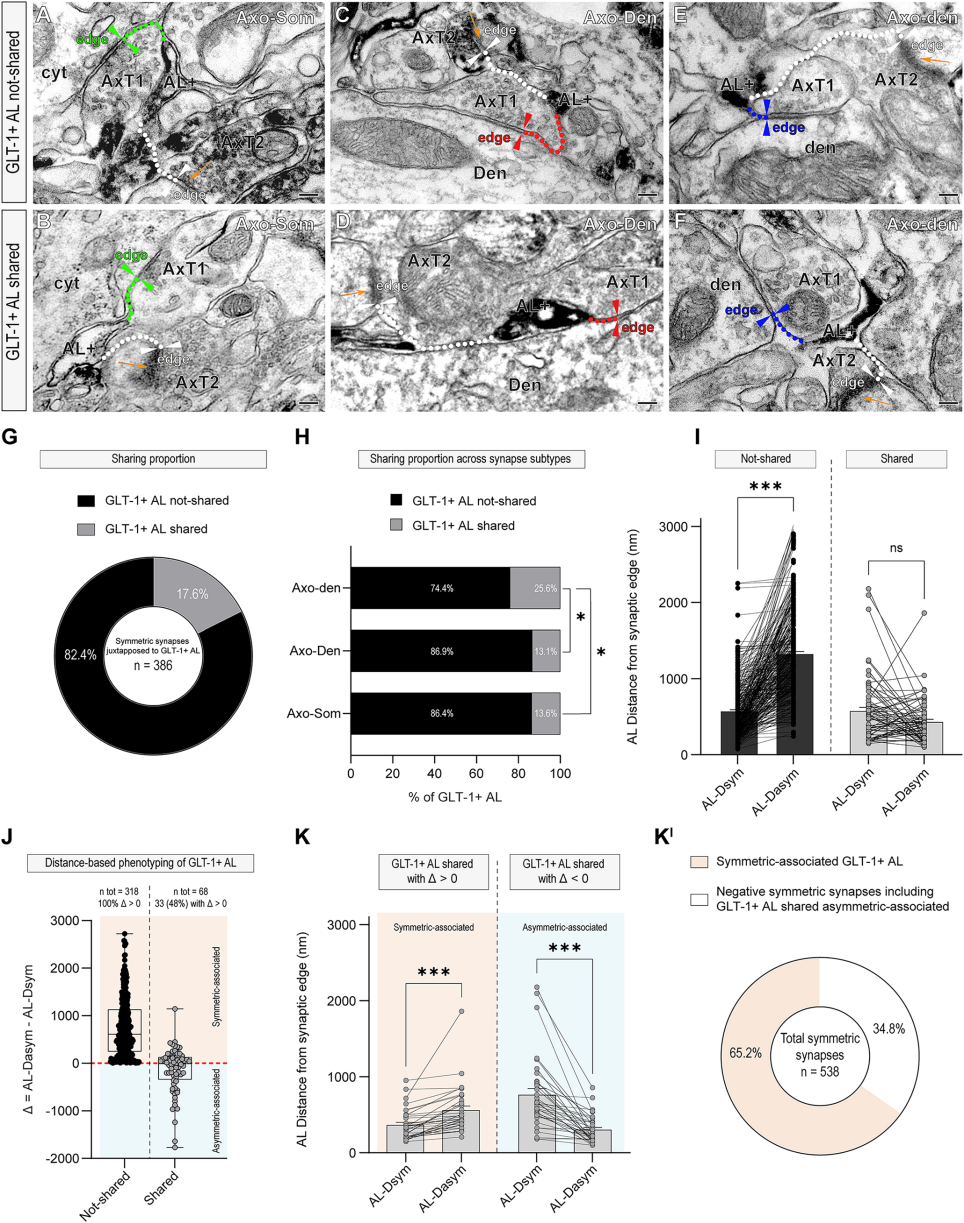
Distance‐based phenotyping of GLT‐1+ ALs juxtaposed to symmetric synapses. (A–F) Representative pre‐embedding electron microscopy illustrating the distance‐based classification of GLT‐1+ ALs at symmetric synapses. (A, C, E) Examples of not‐shared GLT‐1+ ALs in axo‐somatic (A, Axo‐Som), proximal axo‐dendritic (C, Axo‐Den), and distal axo‐dendritic (E, Axo‐den) symmetric synapses, in which the AL is juxtaposed to the symmetric synapse but not to a neighboring asymmetric synapse. (B, D, F) Examples of shared GLT‐1+ ALs in the same synapse subtypes, in which the AL is juxtaposed to both the symmetric synapse and a neighboring asymmetric synapse. Colored and white arrowheads indicate symmetric (AxT1) and asymmetric (AxT2) synaptic edges, respectively; orange arrows indicate neighboring asymmetric synapses. Dotted traces mark the GLT‐1+ AL distances used for distance‐based phenotyping and Δ classification. (G) Of 386 symmetric synapses juxtaposed to GLT‐1+ ALs, 318 were not shared and 68 were shared with a neighboring asymmetric synapse. (H) Composition of GLT‐1+ AL sharing categories across synapse subtypes, showing that the relative proportions of not‐shared and shared ALs differed across subtypes (Axo‐Som: 86.4% not‐shared, 13.6% shared; Axo‐Den: 86.9% not‐shared, 13.1% shared; Axo‐den: 74.4% not‐shared, 25.6% shared; Fisher's exact test, *p* = 0.014; post hoc pairwise comparisons: Axo‐Som vs. Axo‐Den, *p* = 1.000; Axo‐Som vs. Axo‐den, *p* = 0.032; Axo‐Den vs. Axo‐den, *p* = 0.032). (I) Paired comparison of AL‐Dsym and AL‐Dasym in not‐shared and shared GLT‐1+ ALs. In not‐shared profiles, AL‐Dasym was consistently greater than AL‐Dsym (Wilcoxon matched‐pairs signed‐rank test, *p* < 0.001; median difference = 611.7 nm), whereas shared profiles showed no overall difference between the two distances (*p* = 0.112; median difference = −11.11 nm). Points represent individual paired values connected by lines; bars indicate mean ± SEM. (J) Distribution of Δ = AL‐Dasym − AL‐Dsym for not‐shared and shared GLT‐1+ ALs. Not‐shared ALs were exclusively symmetric‐associated (Δ > 0), whereas shared ALs included both symmetric‐associated (Δ > 0) and asymmetric‐associated (Δ < 0) profiles (see also Figure S1A–A′ for scatter plots and synapse subtype‐resolved Δ distributions). Points represent individual Δ values; boxes show the median and interquartile range; whiskers indicate minimum and maximum values. (K) Paired comparison of AL‐Dsym and AL‐Dasym after segregation of shared GLT‐1+ ALs according to the sign of Δ. Shared symmetric‐associated profiles (Δ > 0) showed AL‐Dasym >AL‐Dsym (Wilcoxon matched‐pairs signed‐rank test, *p* < 0.001; median difference = 134.8 nm), whereas shared asymmetric‐associated profiles (Δ < 0) showed AL‐Dasym <AL‐Dsym (*p* < 0.001; median difference = −288.3 nm). Points represent individual paired values connected by lines; bars indicate mean ± SEM. (K^I^) Of the initial 386 symmetric synapses juxtaposed to GLT‐1+ ALs (386/538 total synapses, 71.6%), 351 were classified as symmetric‐associated after Δ‐based phenotyping, refining the estimate of symmetric synapses juxtaposed to GLT‐1+ ALs to 65.2% (351/538). Data were obtained from 12 ultrathin sections per animal, 3 animals. Distances are two‐dimensional estimates measured in single ultrathin sections. Scale bars: 80 nm.

Given the dense packing of excitatory synapses in cortical neuropil (Antón‐Sánchez et al. 2014), we asked how often GLT‐1+ ALs juxtaposed to a symmetric synapse were also juxtaposed to neighboring asymmetric synapses within the same electron microscopical field, allowing us to classify GLT‐1+ ALs as not‐shared or shared (Figure 2A–F). We then asked whether this proportion differed across GABAergic synapse subtypes. Across all GLT‐1+ ALs juxtaposed to symmetric synapses (*n* = 386; Figure 1I), the majority were not‐shared (318/386, 82.4%), whereas shared ALs accounted for 17.6% (68/386; Figure 2G). The overall frequency of not‐shared versus shared GLT‐1+ ALs differed significantly across synapse subtypes (Fisher's exact test, *p* = 0.014). Post hoc pairwise comparisons showed that this effect was driven by Axo‐den synapses, which differed from both Axo‐Som and Axo‐Den synapses (*p* = 0.032 for both comparisons), whereas Axo‐Som and Axo‐Den synapses did not differ from each other (*p* = 1.000; Figure 2H). However, sharing per se does not establish whether a GLT‐1+ AL is preferentially associated with the symmetric synapse or simply lies within reach of a neighboring asymmetric synapse. To address this, we compared, for each GLT‐1+ AL, the distance to the symmetric synaptic edge (AL‐Dsym) with the distance to the nearest asymmetric synaptic edge in the same field (AL‐Dasym; see Materials and Methods). In not‐shared profiles, AL‐Dasym was consistently greater than AL‐Dsym (Wilcoxon matched‐pairs signed‐rank test, *p* < 0.001; median AL‐Dsym = 488 nm, median AL‐Dasym = 1203 nm; Figure 2A,C,E,I, left), indicating a clear preferential association with the symmetric synapse. By contrast, shared profiles showed no overall difference between AL‐Dsym and AL‐Dasym (*p* = 0.112; median AL‐Dsym = 492.1 nm, median AL‐Dasym = 399.3 nm; Figure 2I, right), suggesting that this class comprised heterogeneous spatial configurations. To resolve this heterogeneity, we introduced a distance‐based phenotyping based on Δ = AL‐Dasym − AL‐Dsym (Figure 2J; see Materials and Methods). By this criterion, all not‐shared ALs were classified as symmetric‐associated (Δ > 0). In contrast, shared ALs segregated into symmetric‐associated profiles (Δ > 0; AL‐Dasym > AL‐Dsym; *p* < 0.001; median AL‐Dsym = 333.2 nm, median AL‐Dasym = 477.3 nm; Figure 2B,D,F,J,K, left) and asymmetric‐associated profiles (Δ < 0; AL‐Dasym<AL‐Dsym; *p* < 0.001; median AL‐Dsym = 647.8 nm, median AL‐Dasym = 239.1 nm; Figure 2K, right; Figure S1A–D). Thus, although the frequency of shared GLT‐1+ ALs differed across synapse subtypes, Δ‐based phenotyping revealed that a substantial fraction of shared profiles remained symmetric‐associated. Indeed, within the shared subset, the proportion of symmetric‐associated profiles (Δ > 0) ranged from 23.8% in Axo‐Som (5/21) and 38.5% in Axo‐Den (5/13) to 67.6% in Axo‐den (23/34) (Figure S1A′). Strikingly, once not‐shared and shared profiles were integrated through Δ‐based phenotyping, the total fraction of symmetric‐associated GLT‐1+ ALs remained comparable across Axo‐Som, Axo‐Den, and Axo‐den synapses (Fisher's exact test, *p* = 0.831; Figure S2), thereby refining the estimate of symmetric synapses juxtaposed to GLT‐1+ ALs from 71.6% (386/538) of all symmetric synapses examined (Figure 1I) to 65.2% (351/538) after distance‐based phenotyping (Figure 2K′). Notably, by refining the initial two‐dimensional estimate, the distance‐based phenotyping yielded a value (65.2%) that closely matches that obtained by three‐dimensional electron microscopy for astrocytic contacts at symmetric synapses (approximately 61%; Kikuchi et al. 2020), thus supporting the reliability of the subsequent analyses.

Together, these analyses quantified the extent to which GLT‐1+ ALs were shared between inhibitory and excitatory synapses and established an explicit distance‐based criterion to define a conservative population of symmetric‐associated GLT‐1+ ALs. These findings further indicated that, despite subtype‐specific differences in local sharing, the preferential polarization of GLT‐1+ ALs toward symmetric synapses is broadly conserved across inhibitory synapse subtypes.

### 3.3 Distal Axo‐Dendritic Synapses Are Characterized by Closer GLT‐1+ ALs Embedded in a Tighter Local Excitatory Microenvironment

Having identified the population of symmetric‐associated GLT‐1+ ALs, we next asked whether AL geometry differs across symmetric synapse subtypes and whether it covaries with the local excitatory (i.e., asymmetric) neighborhood.

**FIGURE 3:**
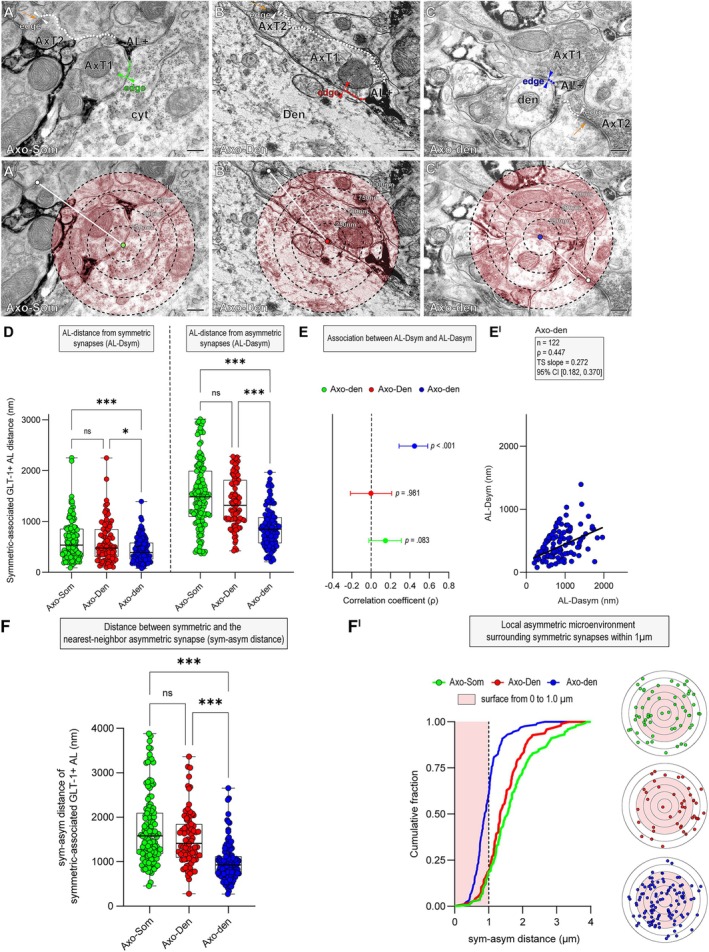
Symmetric‐associated GLT‐1+ ALs distances and local excitatory microenvironment across symmetric synapse subtypes. (A–C) Representative symmetric‐associated GLT‐1+ ALs + at axo‐somatic (A, Axo‐Som), proximal axo‐dendritic (B, Axo‐Den), and distal axo‐dendritic (C, Axo‐den) synapses. As in Figure 2, colored and white arrowheads indicate symmetric (AxT1) and asymmetric (AxT2) synaptic edges, respectively; orange arrows indicate neighboring asymmetric synaptic contacts. Dotted traces mark the AL distances measured from the AL+ to the closest symmetric synaptic edge (AL‐Dsym) and to the nearest asymmetric synaptic edge in the same microscopic field (AL‐Dasym). (A^I^–C^I^) The same fields shown in A–C with schematic radial masks used to adapt a Sholl‐like analysis to the ultrastructural images (for details, see Materials and Methods). White lines represent the Euclidean distance from the center of the symmetric synapse (colored dots) to the nearest asymmetric synapse in the same field (white dots). (D) Distribution of AL‐Dsym (left) and AL‐Dasym (right) across symmetric synapse subtypes. In Axo‐den synapses, AL‐Dsym values were shorter than those of Axo‐Som and Axo‐Den synapses (Kruskal–Wallis H = 14.17, *p* < 0.001; Dunn–Holm: Axo‐Som vs. Axo‐Den, *p* = 1.000; Axo‐Som vs. Axo‐den, *p* < 0.001; Axo‐Den vs. Axo‐den, *p* = 0.035). AL‐Dasym also differed across subtypes (Kruskal–Wallis H = 87.20, *p* < 0.001; Dunn–Holm: Axo‐Som vs. Axo‐Den, *p* = 0.659; Axo‐Som vs. Axo‐den, *p* < 0.001; Axo‐Den vs. Axo‐den, *p* < 0.001). Points represent individual AL distance values; boxes show the median and interquartile range; whiskers indicate minimum and maximum values. (E) Spearman correlation coefficients (*ρ*) for the association between AL‐Dsym and AL‐Dasym in each synapse subtype. A positive monotonic association was significant in Axo‐den (*ρ* = 0.447, *p* < 0.001), but not in Axo‐Som (*ρ* = 0.148, *p* = 0.083) or Axo‐Den (*ρ* = −0.003, *p* = 0.981). (E^I^) Scatter plot of AL‐Dsym versus AL‐Dasym in Axo‐den, with the black line indicating the Theil–Sen fit; the inset reports sample size, Spearman's ρ and the Theil–Sen slope with bootstrap 95% confidence interval. (F–F^I^) Analysis of the local excitatory microenvironment surrounding symmetric synapses. (F) Sym–asym distance across Axo‐Som, Axo‐Den, and Axo‐den synapses. Sym–asym distance differed across subtypes (Kruskal–Wallis H = 91.87, *p* < 0.001; Dunn–Holm: Axo‐Som vs. Axo‐Den, *p* = 0.604; Axo‐Som vs. Axo‐den, *p* < 0.001; Axo‐Den vs. Axo‐den, *p* < 0.001). Points represent individual sym‐asym distance values; boxes show the median and interquartile range; whiskers indicate minimum and maximum values. (F^I^) ECDFs (left) of sym–asym distance (μm), showing closer asymmetric neighbors in Axo‐den than in the other subtypes (Kruskal–Wallis H = 100.84, *p* < 0.001; Dunn–Holm: Axo‐den vs. Axo‐Den, *p* < 0.001; Axo‐den vs. Axo‐Som, *p* < 0.001; Axo‐Den vs. Axo‐Som, *p* = 0.123). The shaded region marks fields with sym–asym distance ≤ 1.0 μm, whose fraction also differed across groups (*χ* ^2^ = 67.061, *p* < 0.001; Fisher–Holm: Axo‐den vs. Axo‐Den, *p* < 0.001; Axo‐den vs. Axo‐Som, *p* < 0.001; Axo‐Den vs. Axo‐Som, *p* = 1.000). Right, radial maps illustrate the position of the nearest asymmetric synapse around symmetric synaptic contacts in the three subtypes. Data were obtained from 12 ultrathin sections per animal, 3 animals. Distances are two‐dimensional estimates measured in single ultrathin sections. Scale bars: 230 nm.

To this end, we first compared AL‐Dsym and AL‐Dasym across symmetric synapse subtypes (Figure 3A–D). Among symmetric synapse subtypes, AL‐Dsym differed significantly (Kruskal–Wallis H = 14.17, *p* < 0.001; median AL‐Dsym: Axo‐Som = 537 nm, Axo‐Den = 477.2 nm, Axo‐den = 388 nm), with Axo‐den synapses showing shorter AL‐Dsym values than Axo‐Som and Axo‐Den synapses (Dunn–Holm: Axo‐Som vs. Axo‐den, *p* < 0.001; Axo‐Den vs. Axo‐den, *p* = 0.035), while Axo‐Som and Axo‐Den were comparable (*p* = 1.000) (Figure 3D, left). This trend was mirrored by AL‐Dasym distances. Indeed AL‐Dasym also differed across subtypes (Kruskal–Wallis H = 87.20, *p* < 0.001; median AL‐Dasym: Axo‐Som = 1483 nm, Axo‐Den = 1320 nm, Axo‐den = 849.1 nm), with Axo‐den synapses showing shorter AL‐Dasym values than both Axo‐Som and Axo‐Den (Dunn–Holm: both *p* < 0.001), whereas Axo‐Som and Axo‐Den did not differ (*p* = 0.659) (Figure 3D, right). We then tested whether symmetric‐ and asymmetric‐referenced distances covary within each symmetric synapse subtype. A significant positive association between AL‐Dsym and AL‐Dasym was observed in Axo‐den synapses (Spearman *ρ* = 0.447, *p* < 0.001), but not in Axo‐Som (*ρ* = 0.148, *p* = 0.083) or Axo‐Den synapses (*ρ* = −0.003, *p* = 0.981) (Figure 3E–E′). Thus, within distal axo‐dendritic synapses, a shorter distance to the nearest asymmetric synapse was associated with a shorter distance of the GLT‐1+ AL from the symmetric synaptic edge.

We further asked whether symmetric synapses juxtaposed to symmetric‐associated GLT‐1+ ALs were embedded in distinct local excitatory microenvironments across inhibitory synapse subtypes. Independently of the AL‐based measurements, we quantified the nearest‐neighbor Euclidean distance between each symmetric synapse and the closest asymmetric synapse in the same field (sym–asym distance; Figure 3A′–C′). Sym–asym distance differed across subtypes (Kruskal–Wallis H = 91.87, *p* < 0.001; median sym–asym distance: Axo‐Som = 1580 nm, Axo‐De*n* = 1411 nm, Axo‐den = 930 nm), with Axo‐den synapses embedded in a closer excitatory microenvironment than Axo‐Som and Axo‐Den (Dunn–Holm: both *p* < 0.001), while Axo‐Som and Axo‐Den were comparable (*p* = 0.604) (Figure 3F). Empirical Cumulative Distribution Functions analyses (Figure 3F′) confirmed a left‐shifted distribution in Axo‐den synapses (Kruskal–Wallis H = 100.84, *p* < 0.001; Dunn–Holm: Axo‐den vs. Axo‐Den, *p* < 0.001; Axo‐den vs. Axo‐Som, *p* < 0.001; Axo‐Den vs. Axo‐Som, *p* = 0.123), and the fraction of fields with sym–asym distance ≤ 1.0 μm differed across groups (χ^2^ = 67.061, *p* < 0.001; Fisher–Holm: Axo‐den vs. Axo‐Den, *p* < 0.001; Axo‐den vs. Axo‐Som, *p* < 0.001; Axo‐Den vs. Axo‐Som, *p* = 1.000) (Figure 3F′).

Together, these analyses showed that Axo‐den synapses were associated with shorter symmetric‐associated GLT‐1+ AL distances and were embedded in a tighter local excitatory microenvironment than Axo‐Som and Axo‐Den synapses. The positive association between AL‐Dsym and AL‐Dasym in Axo‐den synapses further indicated that GLT‐1+ astrocytic positioning might be spatially coordinated with the proximity of neighboring excitatory synapses specifically at distal axo‐dendritic inhibitory contacts.

### 3.4 Membrane‐Associated GLT‐1 Distribution Differs Between Proximal and Distal Symmetric Synapses

**FIGURE 4:**
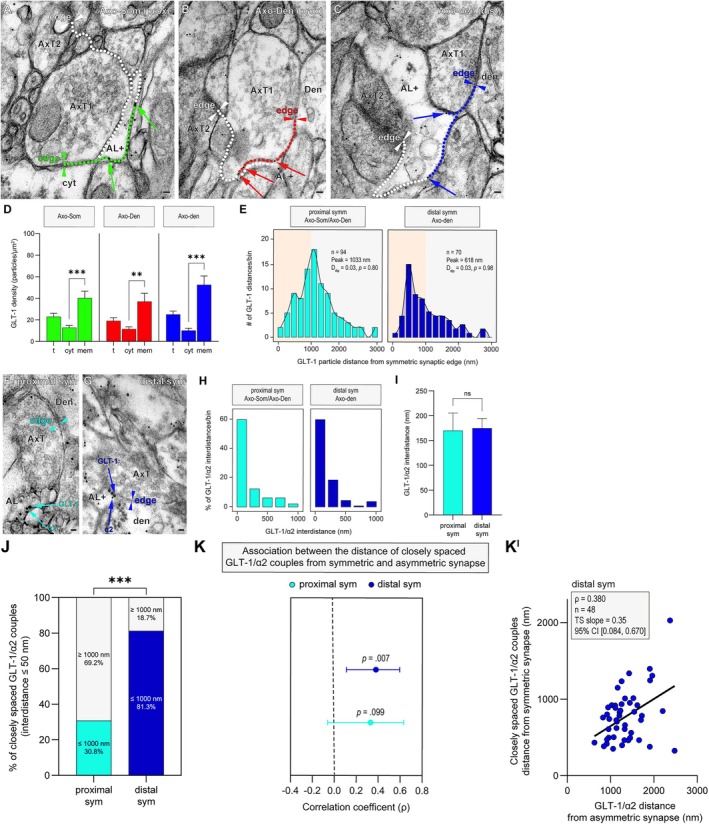
Spatial organization of GLT‐1 and GLT‐1/α2 couples at ALs juxtaposed to symmetric synapses. (A–C) Representative post‐embedding immunogold labeling for GLT‐1 at AL+ juxtaposed to axo‐somatic (A, Axo‐Som), proximal axo‐dendritic (B, Axo‐Den), and distal axo‐dendritic (C, Axo‐den) symmetric synapses. Colored arrows indicate membrane‐associated GLT‐1 gold particles. As in Figure 2, colored and white arrowheads indicate symmetric (AxT1) and asymmetric (AxT2) synaptic edges, respectively; dotted colored and white traces mark the distances of GLT‐1 gold particles from the nearest symmetric and asymmetric synaptic edges, allowing selection of particles closer to symmetric than to asymmetric contacts according to the phenotyping approach used in the pre‐embedding analyses. (D) GLT‐1 immunogold labeling in ALs juxtaposed to symmetric synapses. In all subtypes, GLT‐1 density was higher at the plasma membrane than in the cytoplasm (Axo‐Som, *p* < 0.001; Axo‐Den, *p* = 0.001; Axo‐den, *p* < 0.001; see Table 2a). (E) Distribution of membrane‐associated GLT‐1 particle (selected by Δ‐based phenotyping approach) distances from the edge of proximal (Axo‐Som/Axo‐Den; pooled since the proportion of membrane GLT‐1 particles within 1000 nm from the symmetric synaptic edge did not differ between these two‐subtype, Fisher's exact test, *p* = 0.525; see Table 2b) and distal (Axo‐den) symmetric synapses. Histograms show the number of particles counted in 200‐nm bins along the membrane. Distributions were unimodal in both groups (proximal: Peak = 1033 nm, dip test *p* = 0.806; distal: Peak = 618 nm, dip test *p* = 0.989). The overall distributions differed significantly between groups (two‐sample Kolmogorov–Smirnov test: *D* = 0.216, *p* = 0.039). The proportion of membrane GLT‐1 particles within 1000 nm from the symmetric synaptic edge differed between proximal and distal synapses (Fisher's exact test, *p* = 0.018, see Table 2b). (F–G) Double post‐embedding immunogold labeling for GLT‐1 (18 nm) and α2 (12 nm) in ALs contacting proximal (F) and distal (G) symmetric synapses. Colored arrows indicate representative closely spaced GLT‐1/α2 immunogold couples. In both proximal and distal ALs, membrane‐associated GLT‐1 and α2 densities were higher than cytoplasmic densities (*p* < 0.001 for all comparisons; see Table 2c). (H) Distribution of GLT‐1/α2 interdistances at the membranes of positive astrocytic profiles, shown as 200‐nm bins. In both proximal and distal synapses, the highest proportion of pairs fell within the 0–200 nm bin. A two‐sample Kolmogorov–Smirnov test did not detect a significant difference in distribution shape between groups (*D* = 0.23, *p* = 0.065). (I) GLT‐1/α2 interdistances were comparable between proximal and distal symmetric synapses (proximal and distal interdistances, *n* = 42 and *n* = 118 respectively; Mann–Whitney test, *p* = 0.117). Bars indicate mean ± SEM (proximal 168.88 ± 35.47 nm; distal 174.87 ± 19.30 nm). (J) The proportion of closely spaced GLT‐1/α2 couples (interdistance ≤ 50 nm) located within 1000 nm from the symmetric synaptic edge was higher in distal than in proximal synapses (proximal: 8/26, 30.8%; distal: 39/48, 81.3%; Fisher's exact test, *p* < 0.001). (K) Correlation coefficients for the association between the distances of closely spaced GLT‐1/α2 couples from the symmetric and asymmetric synapses. A positive association was significant in distal synapses (Spearman *ρ* = 0.380, *p* = 0.007), but not in proximal synapses (*ρ* = 0.330, *p* = 0.099). (K^I^) Scatter plot for distal synapses showing the association between the distance of closely spaced GLT‐1/α2 couples from the symmetric synapse and from the neighboring asymmetric synapse. The black line indicates the Theil–Sen fit; the inset reports sample size, Spearman's *ρ* and the Theil–Sen slope with bootstrap 95% confidence interval. Data were obtained from 12 to 15 ultrathin sections per animal, 3 animals. Distances are two‐dimensional estimates measured in single ultrathin sections. Scale bars: 60 nm for A‐C and 35 nm for F‐G.

Because pre‐embedding immunoperoxidase does not resolve intracellular versus membrane‐associated pools, we next used post‐embedding immunogold to directly assess the subcellular compartmentalization and the spatial distribution of membrane‐associated GLT‐1 at ALs juxtaposed to morphologically identified symmetric synapses (Figure 4A–C). Across Axo‐Som, Axo‐Den, and Axo‐den synapse subtypes, GLT‐1 immunogold density in ALs was higher at the plasma membrane than in the cytoplasm (Axo‐Som, *p* < 0.001; Axo‐Den, *p* = 0.001; Axo‐den, *p* < 0.001; Figure 4D; Table 2a).

**TABLE 2a:** Quantification of GLT1 immunogold staining in ALs juxtaposing Axo‐Som, Axo‐Den, Axo‐den symmetric synapses.

Localization	Axo‐Som (particles/μm^2^)	Axo‐Den (particles/μm^2^)	Axo‐den (particles/μm^2^)
Astrocytic processes^a^	22.99 ± 3.08 (*n* = 30)	19.24 ± 2.89 (*n* = 17)	25.23 ± 3.14 (*n* = 35)
Plasma membrane^b^	40.30 ± 6.30	37.20 ± 7.66	52.81 ± 8.19
Cytoplasm	12.91 ± 2.00	11.54 ± 2.20	10.20 ± 2.20

*Note:* Density values are mean ± SEM; *n* = number of profiles; comparison of densities of synaptic astrocytic processes was performed using Mann–Whitney test (data are from 12 to 15 ultrathin sections/animal; 3 animals).

^a^ Density of GLT‐1 gold particles was significantly higher than background (estimated by calculating the density over neuronal nuclei, *n* = 25, 0.42 ± 0.02) in astrocytic processes surrounding symmetric synapses (*p* < 0.001 for all subsets of symmetric synapses).

^b^ Density of membrane‐associated particles was significantly higher than cytoplasm in astrocytic processes (*p* < 0.001 for Axo‐Som, *p* < 0.001 for Axo‐Den, and *p* < 0.001 for Axo‐den).

To quantify membrane‐associated GLT‐1 minimizing ambiguity introduced by nearby asymmetric synapses, for each GLT‐1 particle we measured its distance along the AL membrane to the nearest symmetric synaptic edge and to the nearest asymmetric synaptic edge in the same field and restricted subsequent analyses to particles classified as symmetric‐associated (i.e., closer to the symmetric than to the asymmetric contact, using the same Δ‐based phenotyping approach applied in pre‐embedding analyses; Figure 4A–C; Figure S3). We then examined the distribution of distances of these membrane‐associated GLT‐1 particles from the symmetric synaptic edge. Because the fraction of particles within 1000 nm did not differ between Axo‐Som and Axo‐Den, proximal synapses were pooled for distributional analyses (*p* = 0.525; Figure 4E; Table 2b). In both proximal and distal groups, distributions were unimodal (proximal peak = 1033 nm, dip test *p* = 0.806; distal peak = 618 nm, dip test *p* = 0.989). However, the overall distributions differed significantly between groups (two‐sample Kolmogorov–Smirnov test: *D* = 0.216, *p* = 0.039). In line with this result, distal synapses showed a higher fraction of membrane‐associated GLT‐1 particles within 1000 nm of the symmetric synaptic edge than proximal synapses (Fisher's exact test, *p* = 0.018; Figure 4E; Table 2b).

**TABLE 2b:** Comparison of membrane‐GLT1 in ALs juxtaposing Axo‐Som, Axo‐Den, and Axo‐den symmetric synapses.

GLT‐1 membrane (n of particles 0–3000 nm)	Axo‐Som (proximal) (*n* = 55)	Axo‐Den (proximal) (*n* = 39)	Axo‐den (distal) (*n* = 70)	Comparison
Axo‐Som vs Axo‐Den	< 1000 (*n* = 24) > 1000 (*n* = 31)	< 1000 (*n* = 14) > 1000 (*n* = 25)	—	*p* = 0.525
Proximal vs Distal	Proximal < 1000 (*n* = 38) > 1000 (*n* = 56)		< 1000 (*n* = 42) > 1000 (*n* = 28)	*p* = 0.018

*Note:* Comparison of GLT‐1 frequency distributions was performed using Fisher's exact test.

### 3.5 Closely Spaced GLT‐1/α2 Membrane‐Associated Couples at Symmetric Synapses Are Enriched Within 1000 Nm at Distal Axo‐Dendritic Synapses

Given the functional relevance of astrocytic α2 for GLT‐1‐associated transport complexes (Melone et al. 2019; Pietrobon and Conti 2024), we first quantified by pre‐embedding electron microscopy α2+ ALs juxtaposed to symmetric synapse subtypes, and then we moved to explore post‐embedding GLT‐1/α2 double‐labeled ALs. As a pre‐embedding benchmark, α2‐positive ALs classified as symmetric‐associated by Δ‐based phenotyping showed synapse subtype‐dependent geometry that mirrored that of symmetric‐associated GLT‐1+ ALs, with shorter distances at Axo‐den than at Axo‐Som/Axo‐Den synapses for both AL‐Dsym (Kruskal–Wallis H = 14.25, *p* < 0.001; median AL‐Dsym: Axo‐Som = 580.8 nm, Axo‐Den = 469.2 nm, Axo‐den = 320.8 nm; Dunn–Holm: Axo‐Som vs. Axo‐den, *p* = 0.001; Axo‐Den vs. Axo‐den, *p* = 0.027; Axo‐Som vs. Axo‐Den, *p* = 1.000; Figure S4A–D, left) and AL‐Dasym (Kruskal–Wallis H = 22.12, *p* < 0.001; median AL‐Dasym: Axo‐Som = 1357 nm, Axo‐Den = 1010 nm, Axo‐den = 598.9 nm; Dunn–Holm: Axo‐Som vs. Axo‐den, *p* < 0.001; Axo‐Den vs. Axo‐den, *p* = 0.040; Axo‐Som vs. Axo‐Den, *p* = 0.116; Figure S4A–D, right). In addition, the prevalence of symmetric synapses juxtaposed to symmetric‐associated α2+ ALs was comparable to that measured for GLT‐1+ ALs (α2: 105/180, 58.3% vs. GLT‐1: 351/538, 65.2%; Fisher's exact test, *p* = 0.107; Figure S4E–F).

In post‐embedded double‐labeled ALs at proximal versus distal symmetric synapses (Figure 4F–G), both GLT‐1 and α2 immunogold densities were higher at the plasma membrane than in the cytoplasm (*p* < 0.001 for all comparisons; Figure 4F–G; Table 2c). The amount of AL membrane sampled did not differ between proximal and distal synapses (Mann–Whitney test, *p* = 0.375; median proximal = 0.037, median distal = 0.033); under these conditions, distal synapses showed a higher membrane density of both GLT‐1 (Mann–Whitney test, *p* = 0.033; median proximal = 29.86, median distal = 44.13) and α2 (Mann–Whitney test, *p* < 0.001; median proximal = 28.59, median distal = 51.18) than proximal synapses. We then quantified GLT‐1/α2 interdistances at the AL membrane (Melone et al. 2019). To relate coupling GLT‐1/α2 to the inhibitory synaptic domain while accounting for nearby excitatory contacts, we applied the same Δ‐based phenotyping used throughout the study to GLT‐1/α2 couples (see Materials and Methods) and obtained the symmetric‐associated couples (see Figure S5A–C). The interdistance distributions (summarized in 200‐nm bins) showed the highest proportion of symmetric‐associated couples in the 0–200 nm bin in both proximal and distal synapses (Figure 4H). A two‐sample Kolmogorov–Smirnov test did not detect a significant difference in distribution shape between groups (*D* = 0.23, *p* = 0.065), and interdistances were also comparable by Mann–Whitney test (*p* = 0.117; Figure 4I). Focusing on the subset of closely spaced (≤ 50 nm edge‐to‐edge) symmetric‐associated GLT‐1/α2 couples, distal synapses showed a higher fraction of couples located within 1000 nm from the symmetric synaptic edge compared with proximal synapses (distal: 39/48, 81.3%; proximal: 8/26, 30.8%; Fisher's exact test, *p* < 0.001; Figure 4L; Table 2c). Finally, distances of closely spaced GLT‐1/α2 couples from the symmetric synapse and from the neighboring asymmetric synapse showed a significant positive association in distal synapses (Spearman *ρ* = 0.380, *p* = 0.007), but not in proximal synapses (*ρ* = 0.330, *p* = 0.099) (Figure 4K–K′).

**TABLE 2c:** Quantification of GLT‐1 and α2 immunogold staining in ALs juxtaposing Axo‐proximal (Axo‐Som and Axo‐Den) and distal (Axo‐den) symmetric synapse.

Localization	Proximal symmetric (particles/μm^2^)	Distal symmetric (particles/μm^2^)
Astrocytic processes^a^	GLT‐1; 18.72 ± 2.72 (*n* = 28)	GLT‐1; 27.78 ± 4.43 (*n* = 48)
	α2; 19.82 ± 3.90	α2; 39.83 ± 4.97
Plasma membrane^b^, ^c^	GLT‐1; 34.68 ± 4.13	GLT‐1; 50.65 ± 6.13
	α2; 35.09 ± 5.06	α2; 59.49 ± 5.48
Cytoplasm	GLT‐1; 4.98 ± 1.69	GLT‐1; 2.25 ± 1.07
	α2; 4.59 ± 2.38	α2; 21.45 ± 6.98
GLT‐1/α2 couples (n of couples with an inter‐distance ≤ 50 nm)	< 1000 (*n* = 8)	< 1000 (*n* = 39)
	> 1000 (*n* = 18)	> 1000 (*n* = 9)
	Proximal vs. Distal *p* < 0.001	

*Note:* Density values are mean ± SEM; *n* = number of profiles; comparison of densities of synaptic astrocytic processes was performed using Mann–Whitney test (data are from 12 double‐labeled ultrathin sections/animal; 3 animals).

^a^ Density of GLT‐1 gold particles was significantly higher than background (density over neuronal nuclei, *n* = 10, 0.50 ± 0.05 for GLT‐1, and 0.43 ± 0.04 for α2) in astrocytic processes surrounding symmetric synapses (*p* < 0.001 for all subsets of symmetric synapses).

^b^ Density of membrane‐associated particles was significantly higher than cytoplasm in astrocytic processes (*p* < 0.001 for all subsets of symmetric synapses).

^c^ Mann–Whitney test comparisons of AL membrane sampled for density estimation and for the density of GLT‐1 and α2 between proximal and distal symmetric synapses were reported in the Results section of the manuscript.

Together, post‐embedding data showed that distal symmetric synapses were characterized by a higher local membrane enrichment of GLT‐1 and α2, a closer positioning of membrane‐associated GLT‐1, and a preferential enrichment of closely spaced GLT‐1/α2 astroglial couples near distal inhibitory contacts. Thus, at single‐molecule resolution, post‐embedding immunogold analyses recapitulated and refined the pre‐embedding evidence that GLT‐1+ ALs juxtaposed to distal axo‐dendritic symmetric synapses were more tightly associated with the inhibitory synaptic domain within a local asymmetric synaptic neighborhood.

### 3.6 GLT‐1 Organization in ALs Juxtaposed to Symmetric Synapses Is Conserved in Human Cortex

**FIGURE 5:**
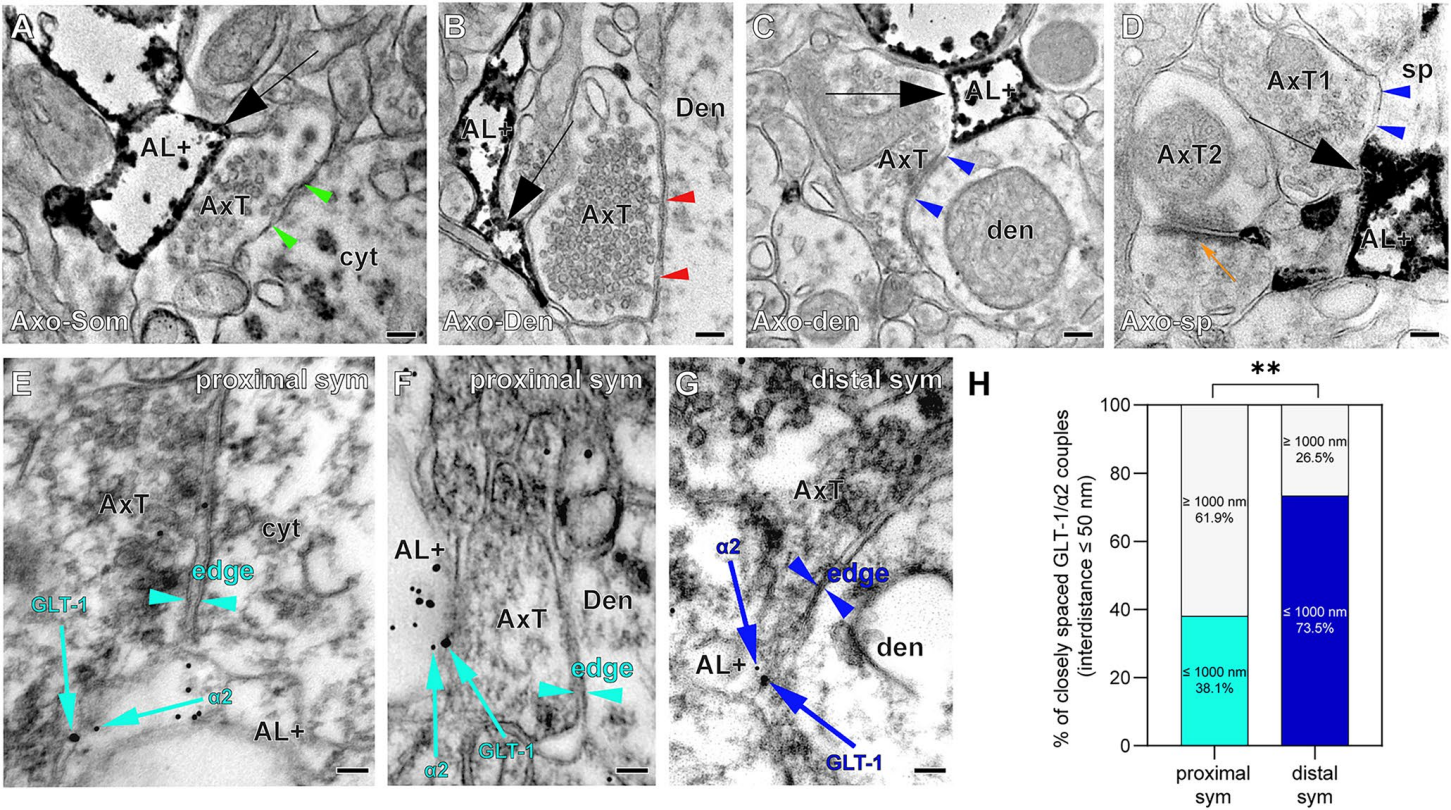
GLT‐1 at ALs juxtaposed to symmetric synapses in human cortex. (A–D) The organization pattern of GLT‐1+ ALs + (pointed by arrows) at proximal (Axo‐Som, Axo‐Den) and distal (Axo‐den, Axo‐sp) symmetric synapses unveiled in rodents is conserved in human cortex (pre‐embedding material of cases HBC 980510, 030129, area 46 and area 8). Colored arrowheads mark the edges of symmetric synaptic contacts; in D, the orange arrow indicates a neighboring asymmetric synapse. (E–G) Double post‐embedding immunogold labeling for GLT‐1 (18 nm) and α2 (12 nm) in ALs juxtaposing proximal (E–F) and distal (G) symmetric synapses from human cortex (cases HBC 980510 and 030129). Light and dark blue arrows indicate closely spaced GLT‐1/α2 immunogold couples. Arrowheads mark the edges of symmetric synaptic contacts. (H) Proportion of closely spaced GLT‐1/α2 couples located within or beyond 1000 nm from the synaptic edge in proximal and distal symmetric synapses. The fraction of couples located within 1000 nm was higher in distal than in proximal synapses (proximal: 8/21, 38.1%; distal: 36/49, 73.5%; Fisher's exact test, *p* = 0.007). Distances are two‐dimensional estimates measured in single ultrathin sections. Scale bars: 140 nm in A–D and 70 nm in E–G.

Examination of human GLT‐1 pre‐embedded processed material (Melone et al. 2011) confirmed the localization of GLT‐1 in ALs juxtaposed to symmetric synapses. In layers II/III of the prefrontal cortex (areas 46 and 8), GLT‐1+ ALs were observed in Axo‐Som, Axo‐Den, and axo‐den symmetric synapses, confirming the expression of GLT‐1 in ALs juxtaposed to these subsets of GABAergic synapses (Figure 5A–D). To assess GLT‐1 membrane‐associated organization, we performed GLT‐1 and α2 double post‐embedding immunogold labeling in human ALs juxtaposed to proximal and distal symmetric synapses (Figure 5E–G). Closely spaced GLT‐1/α2 couples were phenotyped as symmetric‐associated using the same Δ‐based distance criterion applied in the animal dataset (i.e., couples closer to the symmetric than to the neighboring asymmetric synaptic edge within the same field; Figure S5D). In this human dataset, the fraction of symmetric‐associated closely spaced GLT‐1/α2 couples located within 1000 nm from the symmetric synaptic edge was higher at distal than at proximal symmetric synapses (proximal: 8/21, 38.1%; distal: 36/49, 73.5%; Fisher's exact test, *p* = 0.007) (Figure 5H), indicating that the distal enrichment of closely spaced GLT‐1/α2 membrane couples relative to the inhibitory synaptic domain observed in the animal model is conserved in human cortex.

## 4 Discussion

In this study, we combined pre‐embedding immunoperoxidase and post‐embedding immunogold electron microscopy to examine the organization of GLT‐1 at ALs juxtaposed to morphologically identified cortical symmetric synapses. Across inhibitory synapse subtypes, GLT‐1 immunoreactivity was frequently observed in juxtaposed ALs, and distal axo‐dendritic symmetric synapses were characterized by shorter AL‐to‐synaptic‐edge distances and a tighter excitatory nearest‐neighbor context. Pre‐embedding analyses of α2‐positive ALs, consistent with the essential role of α2 in sustaining glutamate transporter cycling, mirrored the symmetric synapse subtype‐dependent geometry of GLT‐1+ ALs, showing that the distal organizational signature was not restricted to GLT‐1 alone. Post‐embedding immunogold further demonstrated that membrane‐associated GLT‐1 at ALs is extrasynaptic relative to symmetric synapses. Closely spaced GLT‐1/α2 couples likewise localized extrasynaptically and showed an enrichment within 1000 nm of symmetric synaptic edges at distal compared with proximal synapses. In addition, distal symmetric synapses showed higher membrane‐associated levels of GLT‐1 and α2 than proximal synapses, strengthening the view that distal inhibitory contacts are associated with a distinct astroglial transporter‐related spatial arrangement. A similar spatial pattern was also observed in human cortex.

The subcellular organization of GLT‐1 at glutamatergic synapses has been extensively described by pre‐ and post‐embedding electron microscopy (Chaudhry et al. 1995; Chen et al. 2004; Furness et al. 2008; Melone et al. 2009, 2011, 2019). In the cerebral cortex, GLT‐1 is expressed in the large majority of excitatory synapses, where it localizes to ALs and axon terminals (Parker et al. 2021). At the membranes of ALs and axon terminals of glutamatergic synapses, GLT‐1 shows a predominant perisynaptic localization (with peaks at 100–150 and 50–100 nm from the AZ edge; Melone et al. 2009, 2011, 2019).

In cortical neuropil, inhibitory synapses are intermingled with a high density of nearby excitatory synapses. Accordingly, we quantified the geometry of GLT‐1+ ALs in relation to the nearest asymmetric synapses, allowing shared and non‐shared configurations to be distinguished and placing inhibitory synapse‐associated GLT‐1 organization within a local excitatory microenvironment (Antón‐Sánchez et al. 2014; Rusakov 2001). Within this context, however, quantitative findings obtained in single ultrathin sections should be interpreted as two‐dimensional estimates rather than absolute three‐dimensional distances. Nonetheless, after Δ‐based phenotyping, symmetric‐associated GLT‐1+ ALs were found at approximately 65% of symmetric synapses, closely matching the ~61% prevalence reported by Kikuchi et al. (2020) in layer IV of rat somatosensory cortex using three‐dimensional electron microscopy. This convergence with recent three‐dimensional data suggests that our two‐dimensional approach captures a biologically meaningful feature of astrocyte‐synapse organization. Accordingly, the Δ‐based estimates should be interpreted as operational upper‐limit estimates of symmetric association, whereas not‐shared ALs define a lower‐limit estimate based on unequivocal configurations. At the same time, the higher prevalence reported here may seem at odds with earlier studies (Cholet et al. 2002) describing glutamate transporter‐immunoreactive ALs as rare near GABAergic elements. However, this apparent discrepancy likely reflects major differences in analytical framework: whereas Cholet et al. (2002) quantified glutamate decarboxylase (GAD)‐positive terminals under highly restrictive “contacted” criteria, the present study focused on ultrastructurally identified symmetric synapses and applied an explicit juxtaposition‐based approach further refined by Δ‐phenotyping. In addition, our Euclidean measurements of the distance from symmetric synapses bearing symmetric‐associated GLT‐1+ ALs to the nearest asymmetric synapse (sym‐asym) were obtained in single ultrathin sections. Therefore, they are not directly equivalent to three‐dimensional centroid‐to‐centroid nearest‐neighbor distances. Nonetheless, the submicron‐to‐low‐micrometer scale of our local excitatory microenvironment measurements (median sym‐asym distance: 1580 nm in Axo‐Som, 1411 nm in Axo‐Den, and 930 nm in Axo‐den synapses) is consistent with independent three‐dimensional ultrastructural estimates of cortical synaptic spacing (Antón‐Sánchez et al. 2014). In particular, Antón‐Sánchez and colleagues reported an overall mean nearest‐neighbor distance of 641.58 nm between synaptic centroids in the same brain region examined here (rat somatosensory cortex), with layer means spanning approximately 555–747 nm, and noted that a substantial fraction of synapses have a neighbor within ≤ 0.5 μm and the large majority within ≤ 1.0 μm. Together, these observations support the plausibility of the local excitatory microenvironment measured here as a two‐dimensional estimate of the synaptic neighborhood surrounding inhibitory synapses. They are also consistent with recent evidence from three‐dimensional electron microscopy and in vivo studies indicating that ALs form interconnected structural units that can enwrap synapses in spatially organized clusters, supporting the “tripartite synaptic network” view (Benoit et al. 2025). Within this framework, the organization of GLT‐1 at inhibitory synapses is best interpreted in the context of the local synaptic neighborhood.

In our dataset, GLT‐1 immunoreactivity at inhibitory synapses was detected in juxtaposed ALs and showed a predominantly extrasynaptic organization relative to symmetric synaptic edges, spanning distances within and beyond 1000 nm at both distal and proximal contacts. This evidence suggests that GLT‐1 may play different roles in modulating glutamate levels at glutamatergic and GABAergic synapses: in the perisynaptic regions of glutamatergic synapses, it regulates glutamate levels close to release sites, shaping glutamatergic transmission and modulating neurotransmitter spillover (Otis et al. 1996; Clements 1996; Diamond and Jahr 1997; Huang and Bergles 2004; Tzingounis and Wadiche 2007), whereas its predominantly extrasynaptic localization near GABAergic synapses may be suited to modulate glutamate diffusion from the extracellular space to inhibitory synapses (“spill‐in”) (Isaacson 2000; Kullmann 2000; Semyanov 2005; Herman et al. 2011; Coddington et al. 2013). Proximal and distal inhibition in cortical neurons is heterogeneous and depends on multiple factors, including interneuron subtype, postsynaptic target location, release properties, receptor composition, and interactions between glutamatergic and GABAergic signaling (Ascoli et al. 2008; Mariotti et al. 2018; Udakis et al. 2020). Electrophysiological studies have shown that glutamate spillover from excitatory synapses can induce significant changes in inhibitory transmission via activation of ionotropic and metabotropic glutamate receptors at or near inhibitory synapses (Min et al. 1999; Carter and Regehr 2000; Mitchell and Silver 2000; Semyanov and Kullmann 2000; Cossart et al. 2000, 2001; Drew et al. 2008; Coddington et al. 2013; Dubois et al. 2016; Mapelli et al. 2016; Mende et al. 2016). Glutamate acting on presynaptic ionotropic receptors can enhance GABA release, whereas activation of mGluRs can reduce inhibitory transmission (van den Pol et al. 1998; Mitchell and Silver 2000; Semyanov and Kullmann 2000; Piet et al. 2003; Scimemi et al. 2004; Pinheiro and Mulle 2008; Drew et al. 2008; Reiner and Levitz 2018; Petroccione et al. ). Consistently, the modulation of inhibitory transmission by glutamate is enhanced by GLT‐1 blockers (Mitchell and Silver 2000; Semyanov and Kullmann 2000; Piet et al. 2003; Scimemi et al. 2004; Drew et al. 2008; Bonfardin et al. 2010), compatible with a scenario in which GLT‐1 positioning at inhibitory synapse‐associated ALs contributes to shaping the glutamate signal reaching GABAergic synapses (Isaacson 2000; Kullmann 2000; Semyanov 2005; Herman et al. 2011; Coddington et al. 2013).

The tighter excitatory nearest‐neighbor context at distal synapses, together with the enrichment of membrane‐associated GLT‐1 and closely spaced GLT‐1/α2 couples, suggests that glutamate spill‐in modulation may be particularly relevant in distal symmetric dendritic domains, where inhibitory synapses coexist with nearby excitatory inputs. Importantly, the larger number of closely spaced GLT‐1/α2 couples near distal inhibitory contacts does not appear to reflect a shift in the intrinsic interdistance of the couples, which was comparable between proximal and distal synapses. Instead, this pattern is consistent with the higher local membrane enrichment of GLT‐1 and α2 observed distally. In this context, the spatial distribution of astrocytic membrane GLT‐1/α2 couples further supports the idea that GLT‐1/α2 organization may vary across synaptic regions of astrocytic processes. This interpretation is consistent with the essential role of α2 in sustaining transporter cycling (Melone et al. 2019; Pietrobon and Conti 2024) and with gradients of glutamate diffusion from synaptic clefts to the extrasynaptic space (Rusakov and Kullmann 1998; Tzingounis and Wadiche 2007; Reiner and Levitz 2018). Differences from previous estimates of α2‐positive astrocytic association at cortical symmetric synapses likely also reflect differences in denominator and unit of analysis. In particular, the fraction reported by Melone et al. (2019) refers to the distribution of α2‐immunoreactive astrocytic profiles across synaptic domains, rather than to the prevalence of α2‐positive ALs across all symmetric synapses, and is therefore not directly comparable to the present synapse‐based estimates. In this respect, the present findings extend previous post‐embedding evidence on GLT‐1/α2 coupling at excitatory synapses (Melone et al. 2019) to the inhibitory synaptic domain, by indicating that, at inhibitory synapses, GLT‐1/α2 coupling microdomains show a preferential distal enrichment. This spatial bias provides a morphological framework for considering differences in local transporter‐associated microdomains around inhibitory synapses.

GLT‐1 at ALs of inhibitory synapses might also play a role in GABA synthesis through the glutamate–glutamine cycle. At inhibitory synapses, GABA is recycled by GATs (Conti et al. 2004; Melone et al. 2015), but decarboxylation of glutamate also contributes to GABA synthesis (Cherubini and Conti 2001; Chaudhry et al. 2002). In GABAergic neurons, one source of glutamate is the neuronal transporter EAAC1, which is expressed in interneurons (Conti et al. 1998, 2011; Danbolt 2001), but the glutamate–glutamine cycle driven by GLT‐1‐mediated glutamate uptake may also contribute (Cherubini and Conti 2001; Chaudhry et al. 2002; Melone et al. 2004, 2006; Liang et al. 2006; Conti et al. 2011), consistent with observations that transporter blockade can abolish glutamate–glutamine cycle‐dependent increases in inhibitory transmission in hippocampal circuits (Liang et al. 2006; Héja et al. 2019).

Finally, in human cortex, pre‐embedding observations and quantitative post‐embedding analyses of closely spaced GLT‐1/α2 couples indicate that the distal enrichment of GLT‐1/α2 couples relative to the inhibitory synaptic domain is conserved, supporting the notion that key organizational features of astroglial transporter positioning at cortical symmetric synapses are shared between rodents and humans.

Overall, the present results support a model in which membrane‐associated GLT‐1 and closely spaced GLT‐1/α2 couples exhibit subtype‐dependent extrasynaptic spatial organization at cortical symmetric synapses, with distal axo‐dendritic synapses characterized by both a tighter excitatory nearest‐neighbor context and a relative enrichment of transporter‐associated membrane proximity within 1000 nm of inhibitory synaptic edges. This structural framework motivates future functional studies testing how local astroglial transporter positioning shapes glutamate dynamics and glutamate‐dependent modulation of inhibitory signaling.

## Author Contributions

**Marcello Melone:** conceptualization, methodology, investigation, formal analysis, data curation, visualization, writing – original draft, writing – review and editing. **Michael Di Palma:** methodology, software, formal analysis, investigation, visualization, writing – review and editing. **Annalisa Scimemi:** writing – review and editing. **Fiorenzo Conti:** conceptualization, supervision, funding acquisition, writing – original draft, writing – review and editing.

## Funding

This work was supported by Italian Ministry of University and Research (grant no. PRIN2022BZWEKA), National Institutes of Health (grant no. R56NS12955601).

## Conflicts of Interest

The authors declare no conflicts of interest.

## Supporting information


**Figure S1:** Distance‐based phenotyping of GLT‐1+ ALs juxtaposed to symmetric synapses. (A) Scatter plot of GLT‐1+ AL distance from the symmetric synaptic edge (AL‐Dsym) versus distance from the nearest asymmetric synaptic edge (AL‐Dasym). The red dashed diagonal indicates AL‐Dasym = AL‐Dsym, namely Δ = AL‐Dasym − AL‐Dsym = 0. Values with AL‐Dasym >AL‐Dsym (Δ > 0) identify symmetric‐associated ALs and are highlighted in pale orange, whereas values with AL‐Dasym <AL‐Dsym (Δ < 0) identify asymmetric‐associated ALs and are highlighted in pale blue. Not‐shared ALs were exclusively symmetric‐associated, whereas shared ALs spanned both categories. (A^I^) Distribution of Δ = AL‐Dasym − AL‐Dsym values for not‐shared and shared ALs across Axo‐Som, Axo‐Den, and Axo‐den symmetric synapses. (B–D) Representative pre‐embedding electron microscopy of symmetric synapses illustrating shared GLT‐1+ AL (AL+) classified as asymmetric‐associated in axo‐somatic (C, Axo‐Som), proximal axo‐dendritic (D, Axo‐Den), and distal axo‐dendritic (E, Axo‐den) symmetric synapses. In each example, the same AL+ is juxtaposed to both the symmetric synapse and a neighboring asymmetric synapse within the same microscopic field. Colored and white arrowheads indicate symmetric (AxT1) and asymmetric (AxT2) synaptic edges, respectively; orange arrows indicate neighboring asymmetric synaptic contacts. Dotted colored and white traces mark the distances from the AL+ to the closest symmetric and asymmetric synaptic edges, used for Δ‐based classification. Because the AL+ was closer to the asymmetric than to the symmetric synapse in these cases, the profiles were classified as asymmetric‐associated. Scale bars: 120 nm.
**Figure S2:** Comparable proportions of symmetric‐associated GLT‐1+ ALs across symmetric synapse subtypes after Δ‐based phenotyping. Stacked bar plots show the distribution of not‐shared GLT‐1+ ALs, shared GLT‐1+ ALs with Δ > 0, and shared GLT‐1+ ALs with Δ < 0 across axo‐somatic (Axo‐Som), proximal axo‐dendritic (Axo‐Den), and distal axo‐dendritic (Axo‐den) symmetric synapses. Percentages are calculated relative to the total number of GLT‐1+ ALs within each synapse subtype. After integration of not‐shared and shared profiles through Δ‐based phenotyping, the proportion of symmetric‐associated GLT‐1+ ALs (not‐shared + shared with Δ > 0) was comparable across subtypes (Axo‐Som, 89.7% (139/155); Axo‐Den, 91.8% (91/99); Axo‐den, 91.7% (122/134); Fisher's exact test, *p* = 0.831).
**Figure S3:** Distance‐based phenotyping of membrane‐associated GLT‐1 immunogold particles relative to symmetric and asymmetric synapses. Paired comparison of distances from single membrane‐associated GLT‐1 particles to the nearest symmetric synaptic edge (particle‐Sym) and to the nearest asymmetric synaptic edge (particle‐Asym) in ALs + juxtaposed to proximal and distal symmetric synapses. In both proximal and distal synapses, particle‐Asym values were significantly greater than particle‐Sym values (proximal: Wilcoxon matched‐pairs signed‐rank test, *p* < 0.001, median difference = 723.1 nm, *n* = 94; distal: *p* < 0.001, median difference = 503.7 nm, *n* = 70).
**Figure S4:** Prevalence and distance‐based phenotyping of symmetric‐associated α2+ ALs juxtaposed to symmetric synapses. (A–C) Representative pre‐embedding electron micrographs of α2‐positive ALs (AL+) juxtaposed to axo‐somatic (A, Axo‐Som; cyt, neuronal cytoplasm), proximal axo‐dendritic (B, Axo‐Den; Den, proximal dendrites), and distal axo‐dendritic (C, Axo‐den; den, distal dendrites) symmetric synapses. Colored and white arrowheads indicate symmetric (AxT1) and asymmetric (AxT2) synaptic edges, respectively; orange arrows indicate neighboring asymmetric synaptic contacts. Dotted colored and white traces mark the distances from α2+ ALs to the closest symmetric and asymmetric synaptic edges, used for distance‐based phenotyping. (D) Distribution of AL‐Dsym (left) and AL‐Dasym (right) for symmetric‐associated α2+ ALs across synapse subtypes. AL‐Dsym differed across subtypes (Kruskal–Wallis H = 14.25, *p* < 0.001; Dunn–Holm: Axo‐Som vs. Axo‐Den, *p* = 1.000; Axo‐Som vs. Axo‐den, *p* = 0.001; Axo‐Den vs. Axo‐den, *p* = 0.027), with shorter values in Axo‐den than in Axo‐Som and Axo‐Den synapses. AL‐Dasym also differed across subtypes (Kruskal–Wallis H = 22.12, *p* < 0.001; Dunn–Holm: Axo‐Som vs. Axo‐Den, *p* = 0.116; Axo‐Som vs. Axo‐den, *p* < 0.001; Axo‐Den vs. Axo‐den, *p* = 0.040), again with shorter values in Axo‐den than in the other subtypes. Points represent individual α2+ AL distance values; boxes show the median and interquartile range; whiskers indicate minimum and maximum values. (E–F) Proportion of symmetric synapses juxtaposed to symmetric‐associated α2+ ALs (E) and GLT‐1+ ALs (F). The prevalence of symmetric‐associated positive ALs was comparable between α2 and GLT‐1 labeling (α2: 105/180, 58.3%; GLT‐1: 351/538, 65.2%; Fisher's exact test, *p* = 0.107). α2 data were obtained from 10 to 12 ultrathin sections per animal, 3 animals. Distances are two‐dimensional estimates measured in single ultrathin sections. Scale bars: 80 nm.
**Figure S5:** Distance‐based phenotyping of membrane‐associated GLT‐1/α2 immunogold couples relative to symmetric and asymmetric synapses. (A, B) Representative double post‐embedding immunogold micrographs showing GLT‐1 (18 nm) and α2 (12 nm) labeling in ALs juxtaposed to proximal (A) and distal (B) symmetric synapses. The larger microscopic fields include both the symmetric synapse (AxT1) and the nearest neighboring asymmetric synapse (AxT2). Colored and white arrowheads indicate symmetric and asymmetric synaptic edges, respectively. Dotted traces mark the distances from the GLT‐1/α2 couples to the nearest symmetric and asymmetric synaptic edges used for distance‐based phenotyping. (C) Paired comparison of distances from all membrane‐associated GLT‐1/α2 immunogold couples (interdistance ≥ and ≤ 50 nm) to the nearest symmetric synaptic edge (particle‐Sym) and to the nearest asymmetric synaptic edge (particle‐Asym) in symmetric‐associated proximal and distal synapses. In both proximal and distal synapses, particle‐Asym values were significantly greater than particle‐Sym values (proximal: Wilcoxon matched‐pairs signed‐rank test, *p* < 0.001, median difference = 636.7 nm, *n* = 118; distal: *p* < 0.001, median difference = 674.8 nm, *n* = 42). (D) Paired comparison of distances from closely spaced GLT‐1/α2 immunogold couples (with interdistance ≤ 50 nm) to the nearest symmetric synaptic edge (particle‐Sym) and to the nearest asymmetric synaptic edge (particle‐Asym) in human proximal and distal symmetric synapses. In both proximal and distal synapses, particle‐Asym values were significantly greater than particle‐Sym values (proximal: Wilcoxon matched‐pairs signed‐rank test, *p* < 0.001, median difference = 424.0 nm, *n* = 21; distal: *p* < 0.001, median difference = 347.0 nm, *n* = 49). Points represent individual paired values connected by lines; bars indicate mean ± SEM. Scale bars: 35 nm.

## Data Availability

The data that support the findings of this study are available on request from the corresponding author. The data are not publicly available due to privacy or ethical restrictions.
