# Supplementary material for: Organization of Astrocytic GLT‐1 at Cortical Inhibitory Synapses
Source: Glia. 2026 Jun 7;74(8):e70180. doi: 10.1002/glia.70180 (PMC13243723; doi:10.1002/glia.70180)
Supplement: Supplementary file 1 — Figure S1: Distance‐based phenotyping of GLT‐1+ ALs juxtaposed to symmetric synapses. (A) Scatter plot of GLT‐1+ AL distance from the symmetric synaptic edge (AL‐Dsym) versus distance from the nearest asymmetric synaptic edge (AL‐Dasym). The red dashed diagonal indicates AL‐Dasym = AL‐Dsym, namely Δ = AL‐Dasym − AL‐Dsym = 0. Values with AL‐Dasym >AL‐Dsym (Δ > 0) identify symmetric‐associated ALs and are highlighted in pale orange, whereas values with AL‐Dasym <AL‐Dsym (Δ < 0) identify asymmetric‐associated ALs and are highlighted in pale blue. Not‐shared ALs were exclusively symmetric‐associated, whereas shared ALs spanned both categories. (AI) Distribution of Δ = AL‐Dasym − AL‐Dsym values for not‐shared and shared ALs across Axo‐Som, Axo‐Den, and Axo‐den symmetric synapses. (B–D) Representative pre‐embedding electron microscopy of symmetric synapses illustrating shared GLT‐1+ AL (AL+) classified as asymmetric‐associated in axo‐somatic (C, Axo‐Som), proximal axo‐dendritic (D, Axo‐Den), and distal axo‐dendritic (E, Axo‐den) symmetric synapses. In each example, the same AL+ is juxtaposed to both the symmetric synapse and a neighboring asymmetric synapse within the same microscopic field. Colored and white arrowheads indicate symmetric (AxT1) and asymmetric (AxT2) synaptic edges, respectively; orange arrows indicate neighboring asymmetric synaptic contacts. Dotted colored and white traces mark the distances from the AL+ to the closest symmetric and asymmetric synaptic edges, used for Δ‐based classification. Because the AL+ was closer to the asymmetric than to the symmetric synapse in these cases, the profiles were classified as asymmetric‐associated. Scale bars: 120 nm. Figure S2: Comparable proportions of symmetric‐associated GLT‐1+ ALs across symmetric synapse subtypes after Δ‐based phenotyping. Stacked bar plots show the distribution of not‐shared GLT‐1+ ALs, shared GLT‐1+ ALs with Δ > 0, and shared GLT‐1+ ALs with Δ < 0 across axo‐somatic (Axo‐Som), proximal axo‐de [file GLIA-74-0-s001.docx]

**Supplementary Information**

**Organization of astrocytic GLT-1 at cortical inhibitory synapses**

Marcello Melone^1,2^, Michael Di Palma^1^, Annalisa Scimemi^3^, Fiorenzo Conti^1,2°^

^1^Section of Neuroscience and Cell Biology, Department of Experimental and Clinical Medicine, Università Politecnica delle Marche, Ancona 60026, Italy; ^2^Center for Neurobiology of Aging, IRCCS INRCA, Ancona 60020, Italy; and ^3^Department of Biology, SUNY Albany, 1400 Washington Avenue, Albany (NY), USA.

**Correspondence:** Fiorenzo Conti [f.conti@univpm.it](mailto:f.conti@univpm.it)

Department of Experimental and Clinical Medicine, Università Politecnica delle Marche, Via Tronto 10/A, 60026 Torrette di Ancona, Ancona (Italy)

°Present address: European Brain Research Institute (EBRI) Rita Levi-Montalcini, Viale Regina Elena 295, 00161 Rome Italy


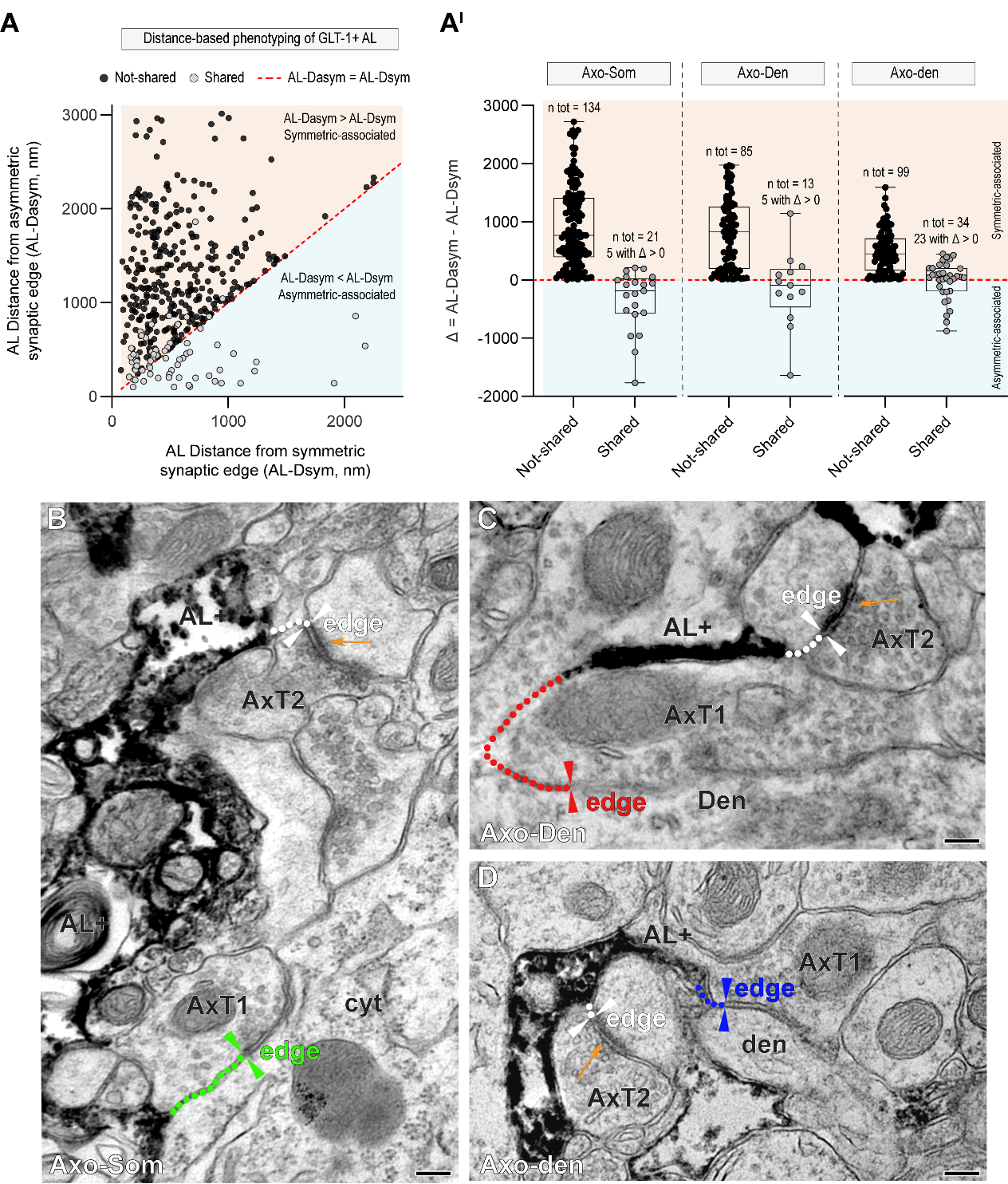


**Supplementary Figure 1. Distance-based phenotyping of GLT-1+ ALs juxtaposed to symmetric synapses.** (A) Scatter plot of GLT-1+ AL distance from the symmetric synaptic edge (AL-Dsym) versus distance from the nearest asymmetric synaptic edge (AL-Dasym). The red dashed diagonal indicates AL-Dasym = AL-Dsym, namely Δ = AL-Dasym − AL-Dsym = 0. Values with AL-Dasym > AL-Dsym (Δ > 0) identify symmetric-associated ALs and are highlighted in pale orange, whereas values with AL-Dasym < AL-Dsym (Δ < 0) identify asymmetric-associated ALs and are highlighted in pale blue. Not-shared ALs were exclusively symmetric-associated, whereas shared ALs spanned both categories. (A^I^) Distribution of Δ = AL-Dasym − AL-Dsym values for not-shared and shared ALs across Axo-Som, Axo-Den, and Axo-den symmetric synapses. (B–D) Representative pre-embedding electron microscopy of symmetric synapses illustrating shared GLT-1+ AL (AL+) classified as asymmetric-associated in axo-somatic (C, Axo-Som), proximal axo-dendritic (D, Axo-Den), and distal axo-dendritic (E, Axo-den) symmetric synapses. In each example, the same AL+ is juxtaposed to both the symmetric synapse and a neighboring asymmetric synapse within the same microscopic field. Colored and white arrowheads indicate symmetric (AxT1) and asymmetric (AxT2) synaptic edges, respectively; orange arrows indicate neighboring asymmetric synaptic contacts. Dotted colored and white traces mark the distances from the AL+ to the closest symmetric and asymmetric synaptic edges, used for Δ-based classification. Because the AL+ was closer to the asymmetric than to the symmetric synapse in these cases, the profiles were classified as asymmetric-associated. Scale bars: 120 nm.


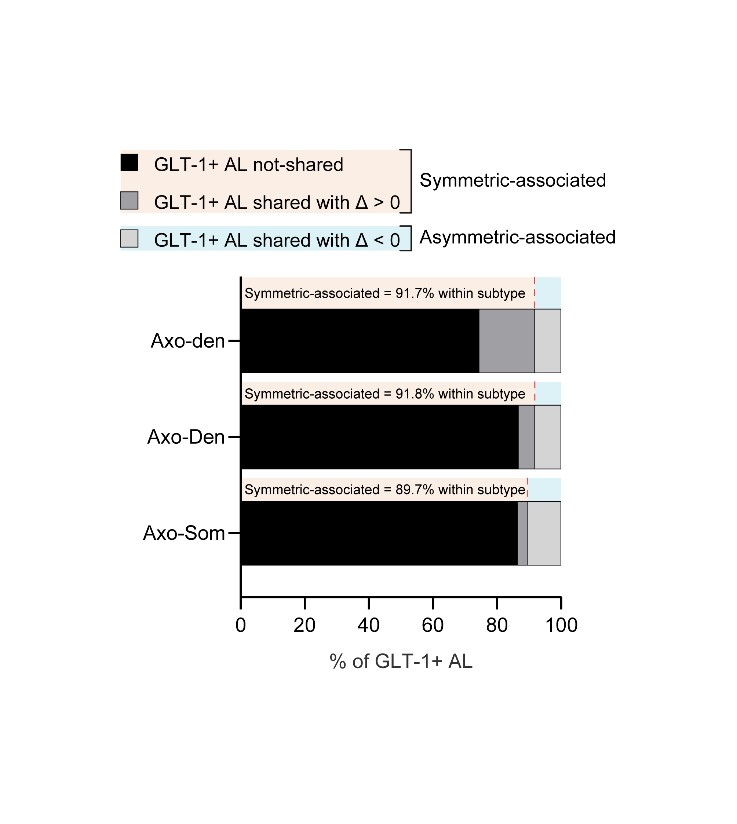


**Supplementary Figure 2. Comparable proportions of symmetric-associated GLT-1+ ALs across symmetric synapse subtypes after Δ-based phenotyping.** Stacked bar plots show the distribution of not-shared GLT-1+ ALs, shared GLT-1+ ALs with Δ > 0, and shared GLT-1+ ALs with Δ < 0 across axo-somatic (Axo-Som), proximal axo-dendritic (Axo-Den), and distal axo-dendritic (Axo-den) symmetric synapses. Percentages are calculated relative to the total number of GLT-1+ ALs within each synapse subtype. After integration of not-shared and shared profiles through Δ-based phenotyping, the proportion of symmetric-associated GLT-1+ ALs (not-shared + shared with Δ > 0) was comparable across subtypes (Axo-Som, 89.7% (139/155); Axo-Den, 91.8% (91/99); Axo-den, 91.7% (122/134); Fisher’s exact test, *p* = .831).


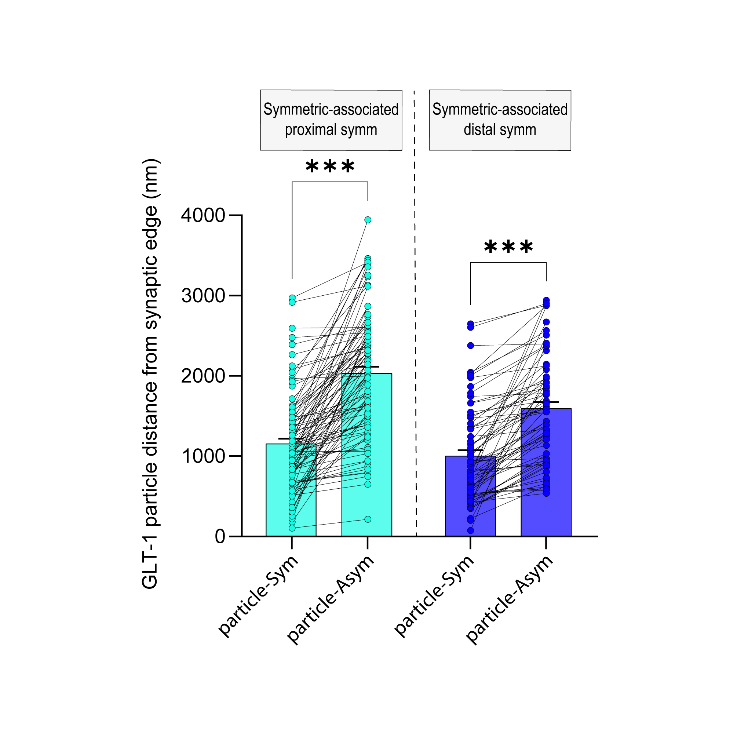


**Supplementary Figure 3. Distance-based phenotyping of membrane-associated GLT-1 immunogold particles relative to symmetric and asymmetric synapses.** Paired comparison of distances from single membrane-associated GLT-1 particles to the nearest symmetric synaptic edge (particle-Sym) and to the nearest asymmetric synaptic edge (particle-Asym) in ALs+ juxtaposed to proximal and distal symmetric synapses. In both proximal and distal synapses, particle-Asym values were significantly greater than particle-Sym values (proximal: Wilcoxon matched-pairs signed-rank test, *p* < .001, median difference = 723.1 nm, n = 94; distal: *p* < .001, median difference = 503.7 nm, n = 70).


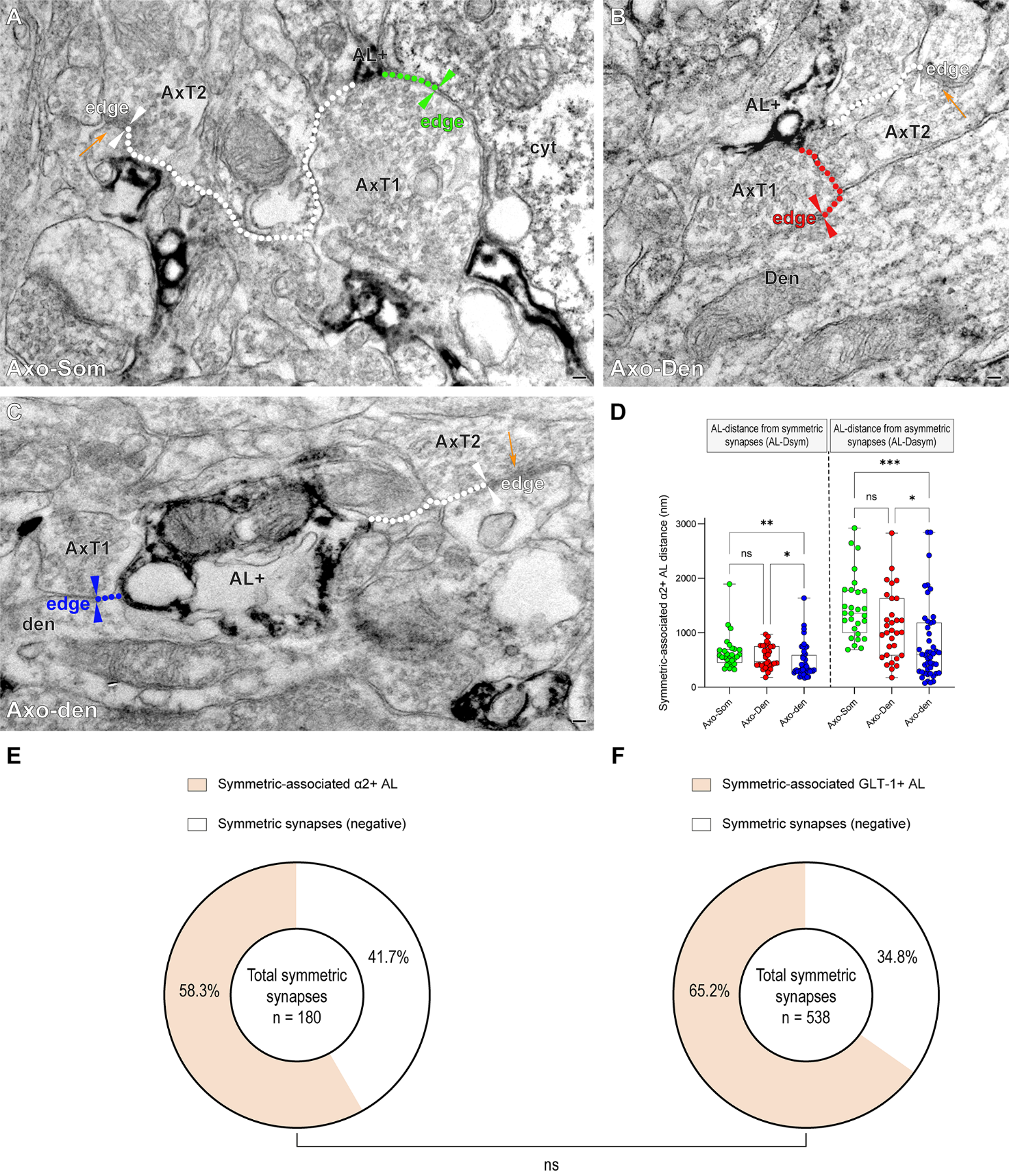


**Supplementary Figure 4. Prevalence and distance-based phenotyping of symmetric-associated α2+ ALs juxtaposed to symmetric synapses.** (A–C) Representative pre-embedding electron micrographs of α2-positive ALs (AL+) juxtaposed to axo-somatic (A, Axo-Som; cyt, neuronal cytoplasm), proximal axo-dendritic (B, Axo-Den; Den, proximal dendrites), and distal axo-dendritic (C, Axo-den; den, distal dendrites) symmetric synapses. Colored and white arrowheads indicate symmetric (AxT1) and asymmetric (AxT2) synaptic edges, respectively; orange arrows indicate neighboring asymmetric synaptic contacts. Dotted colored and white traces mark the distances from α2+ ALs to the closest symmetric and asymmetric synaptic edges, used for distance-based phenotyping. (D) Distribution of AL-Dsym (left) and AL-Dasym (right) for symmetric-associated α2+ ALs across synapse subtypes. AL-Dsym differed across subtypes (Kruskal–Wallis H = 14.25, *p* < .001; Dunn–Holm: Axo-Som vs Axo-Den, *p* = 1.000; Axo-Som vs Axo-den, *p* = .001; Axo-Den vs Axo-den, *p* = .027), with shorter values in Axo-den than in Axo-Som and Axo-Den synapses. AL-Dasym also differed across subtypes (Kruskal–Wallis H = 22.12, *p* < .001; Dunn–Holm: Axo-Som vs Axo-Den, *p* = .116; Axo-Som vs Axo-den, *p* < .001; Axo-Den vs Axo-den, *p* = .040), again with shorter values in Axo-den than in the other subtypes. Points represent individual α2+ AL distance values; boxes show the median and interquartile range; whiskers indicate minimum and maximum values. (E–F) Proportion of symmetric synapses juxtaposed to symmetric-associated α2+ ALs (E) and GLT-1+ ALs (F). The prevalence of symmetric-associated positive ALs was comparable between α2 and GLT-1 labeling (α2: 105/180, 58.3%; GLT-1: 351/538, 65.2%; Fisher’s exact test, *p* = .107). α2 data were obtained from 10–12 ultrathin sections per animal, 3 animals. Distances are two-dimensional estimates measured in single ultrathin sections. Scale bars: 80 nm.


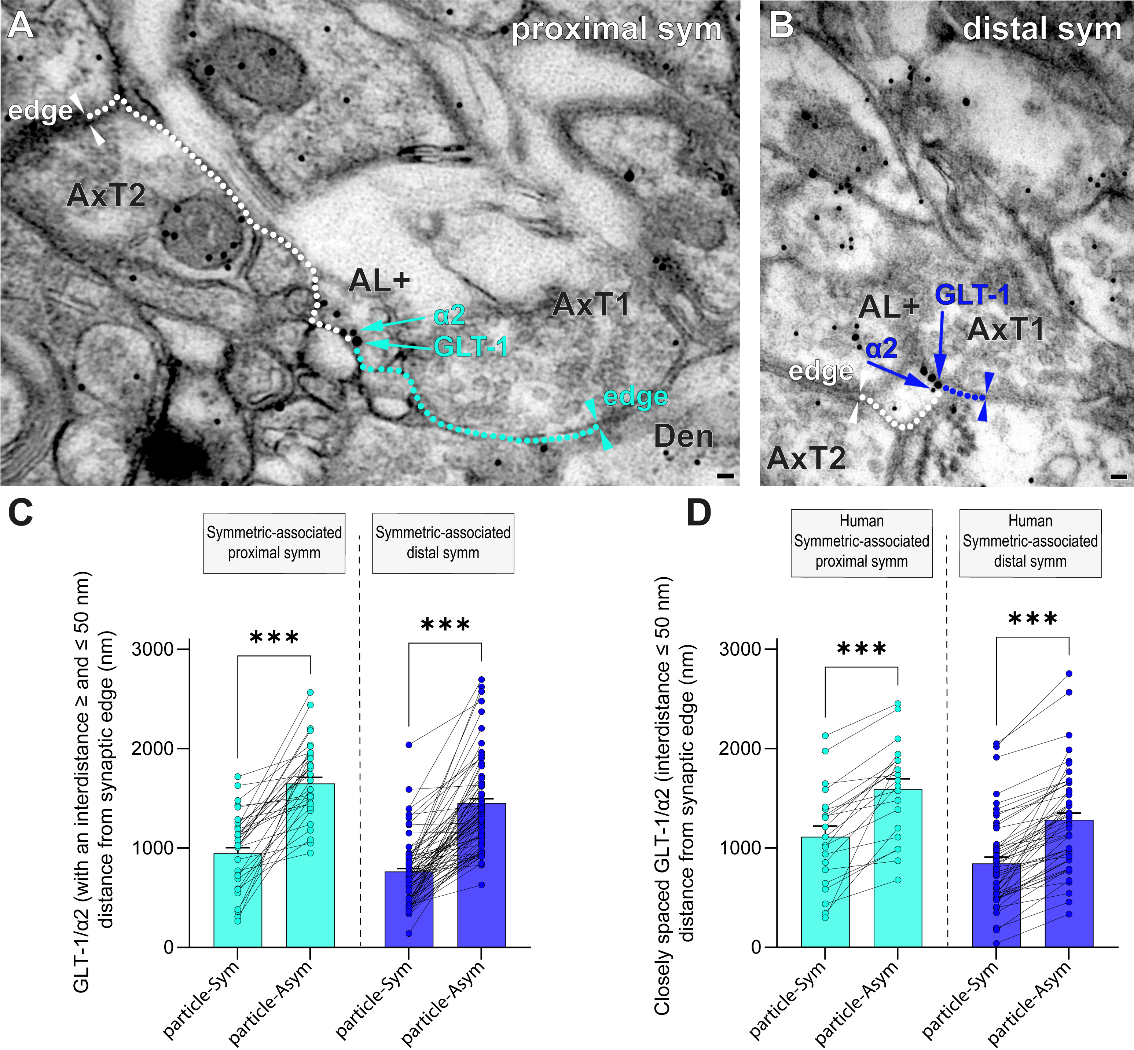


**Supplementary Figure 5. Distance-based phenotyping of membrane-associated GLT-1/α2 immunogold couples relative to symmetric and asymmetric synapses.** (A, B) Representative double post-embedding immunogold micrographs showing GLT-1 (18 nm) and α2 (12 nm) labeling in ALs juxtaposed to proximal (A) and distal (B) symmetric synapses. The larger microscopic fields include both the symmetric synapse (AxT1) and the nearest neighboring asymmetric synapse (AxT2). Colored and white arrowheads indicate symmetric and asymmetric synaptic edges, respectively. Dotted traces mark the distances from the GLT-1/α2 couples to the nearest symmetric and asymmetric synaptic edges used for distance-based phenotyping. (C) Paired comparison of distances from all membrane-associated GLT-1/α2 immunogold couples (interdistance ≥ and ≤ 50 nm) to the nearest symmetric synaptic edge (particle-Sym) and to the nearest asymmetric synaptic edge (particle-Asym) in symmetric-associated proximal and distal synapses. In both proximal and distal synapses, particle-Asym values were significantly greater than particle-Sym values (proximal: Wilcoxon matched-pairs signed-rank test, *p* < .001, median difference = 636.7 nm, n = 118; distal: *p* < .001, median difference = 674.8 nm, n = 42). (D) Paired comparison of distances from closely spaced GLT-1/α2 immunogold couples (with interdistance ≤ 50 nm) to the nearest symmetric synaptic edge (particle-Sym) and to the nearest asymmetric synaptic edge (particle-Asym) in human proximal and distal symmetric synapses. In both proximal and distal synapses, particle-Asym values were significantly greater than particle-Sym values (proximal: Wilcoxon matched-pairs signed-rank test, *p* < .001, median difference = 424.0 nm, n = 21; distal: *p* < .001, median difference = 347.0 nm, n = 49). Points represent individual paired values connected by lines; bars indicate mean ± SEM. Scale bars: 35 nm.
